# Supplementary figures and images for: Antifungal Tolerance and Resistance Emerge at Distinct Drug Concentrations and Rely upon Different Aneuploid Chromosomes
Source: mBio. 2023 Mar 6;14(2):e00227-23. doi: 10.1128/mbio.00227-23 (PMC10127634; doi:10.1128/mbio.00227-23)

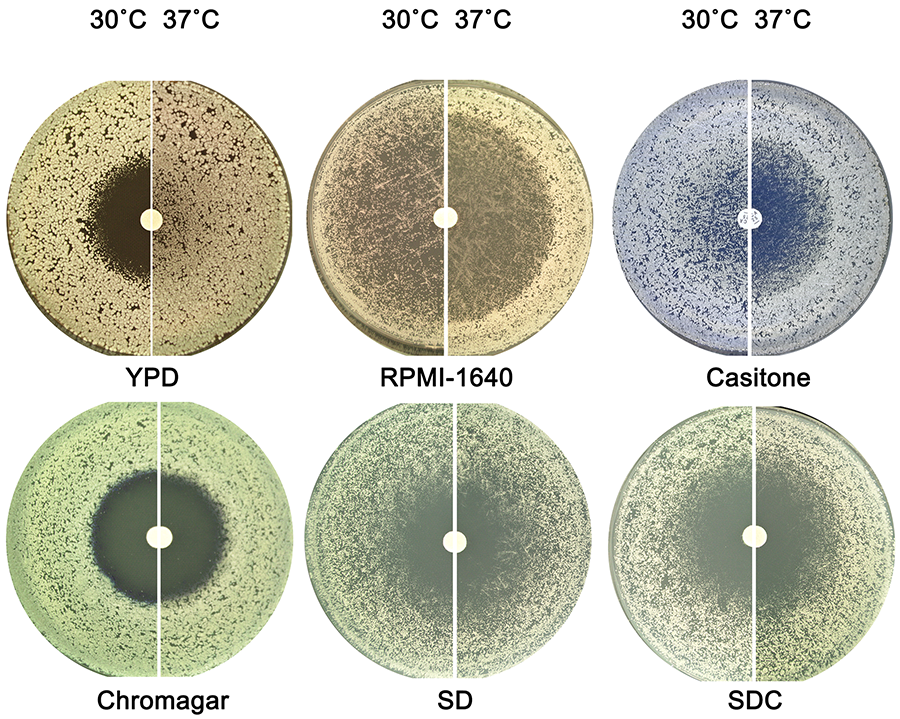

Supplement: FIG S1 [file mbio.00227-23-s0005.tif]

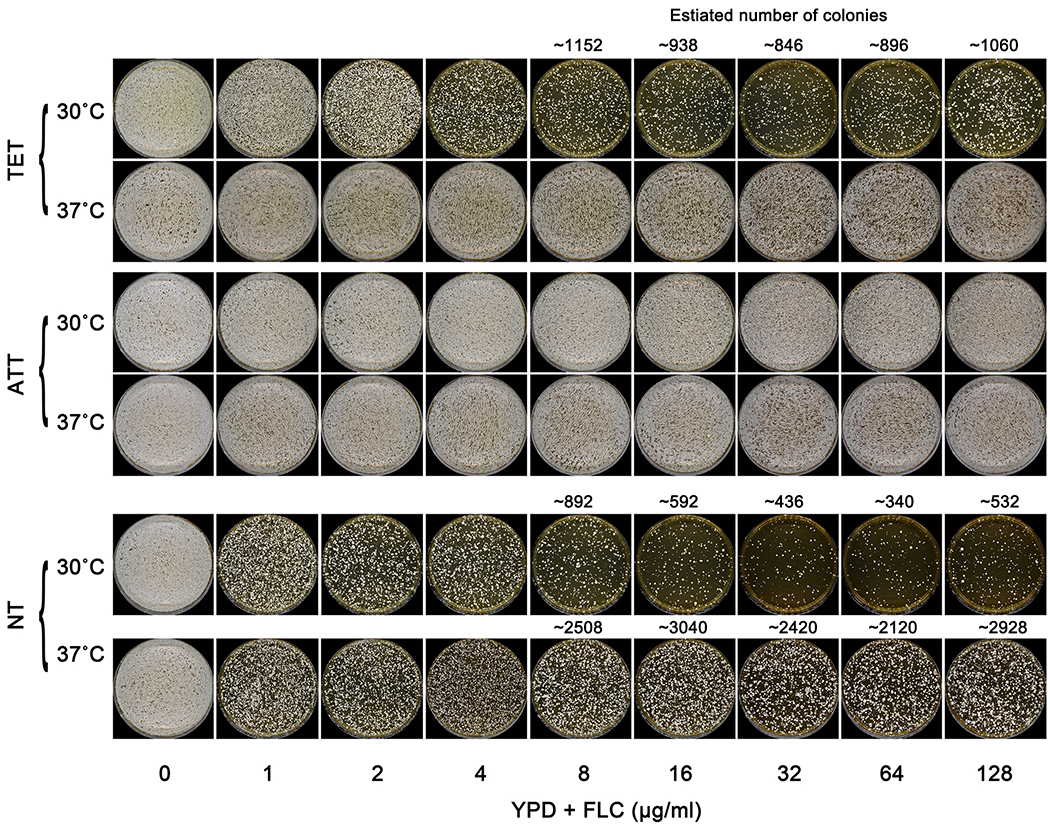

Supplement: FIG S2 [file mbio.00227-23-s0006.tif]

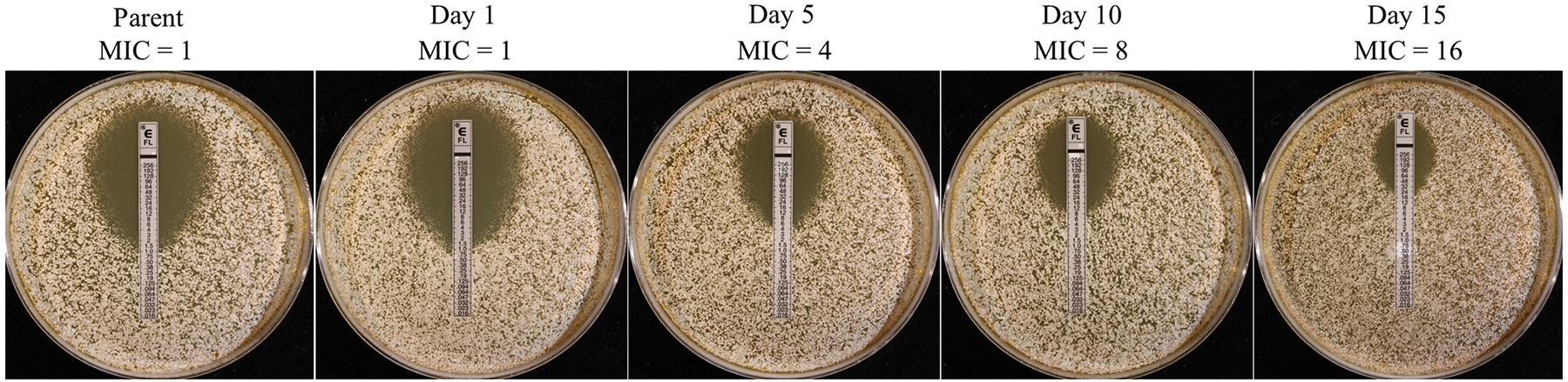

Supplement: FIG S5 [file mbio.00227-23-s0009.tif]
