# Supplementary material for: Antifungal Tolerance and Resistance Emerge at Distinct Drug Concentrations and Rely upon Different Aneuploid Chromosomes
Source: mBio. 2023 Mar 6;14(2):e00227-23. doi: 10.1128/mbio.00227-23 (PMC10127634; doi:10.1128/mbio.00227-23)
Supplement: FIG S3 [file mbio.00227-23-s0007.pdf]

Adaptors derived from SC5314 at 30˚C (n=53)

Adaptors obtained from 8 μg/ml FLC plate (n=17)

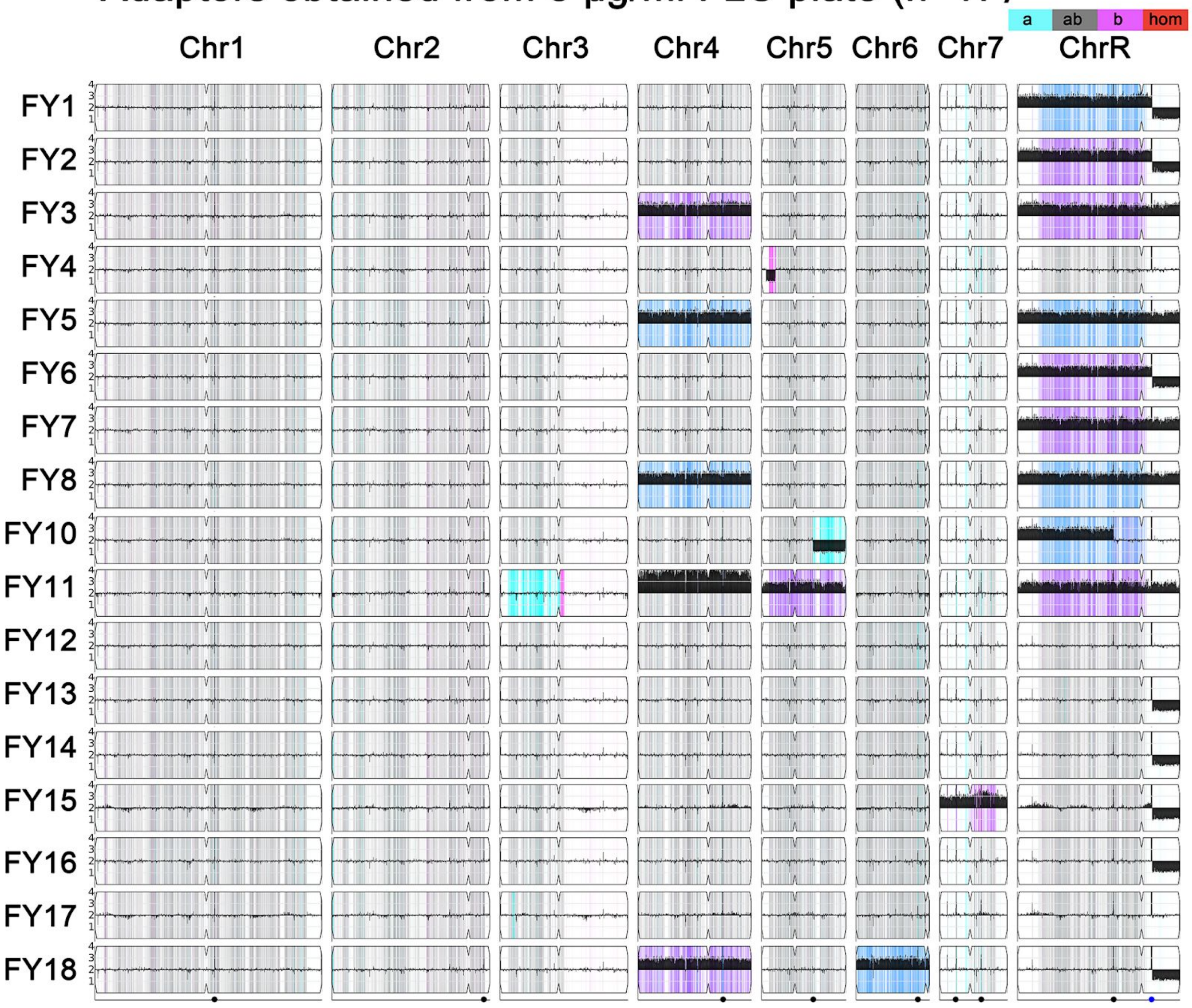

Adaptors obtained from 32 µg/ml FLC plate (n=18)

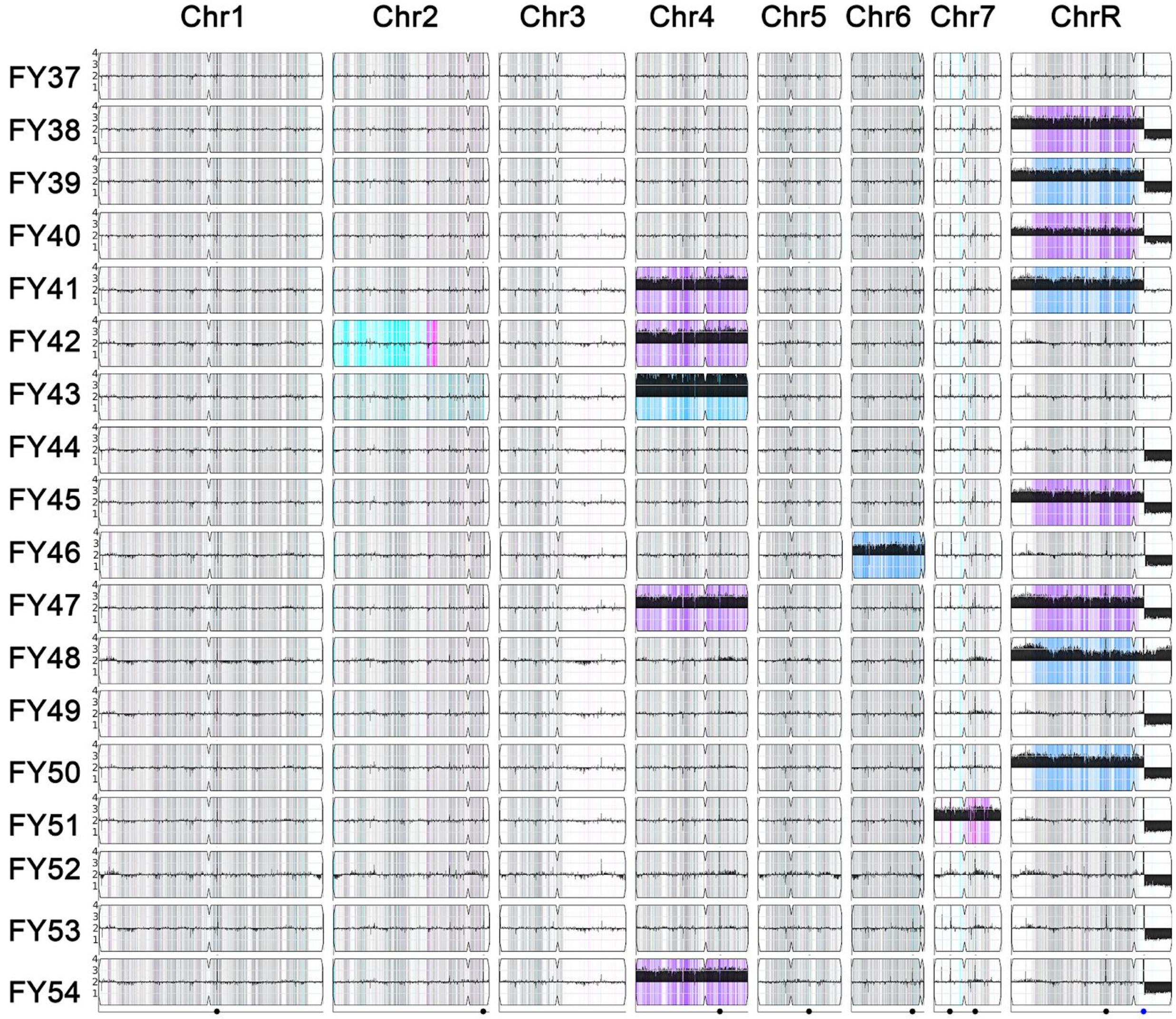

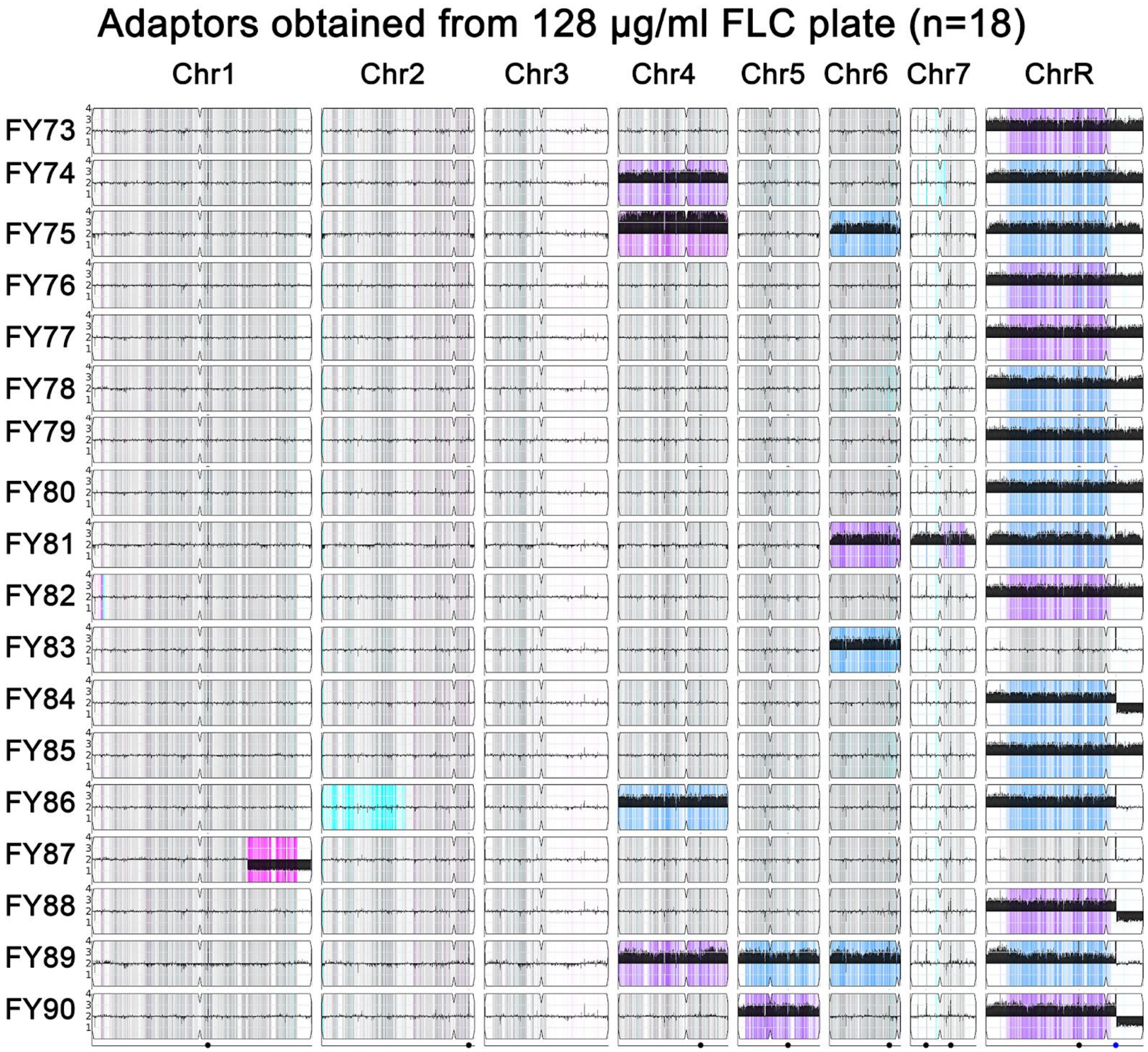

Adaptors obtained from 128 µg/ml FLC plate (n=18)
Chr1
Chr2
Chr3
Chr4
Chr5
Chr6
Chr7
ChrR
FY73
FY74
FY75
FY76
FY77
FY78
FY79
FY80
FY81
FY82
FY83
FY84
FY85
FY86
FY87
FY88
FY89
FY90
