# Supplementary material for: Antifungal Tolerance and Resistance Emerge at Distinct Drug Concentrations and Rely upon Different Aneuploid Chromosomes
Source: mBio. 2023 Mar 6;14(2):e00227-23. doi: 10.1128/mbio.00227-23 (PMC10127634; doi:10.1128/mbio.00227-23)

Adaptors derived from YJBT490 at 30˚C (n=54)

Adaptors obtained from 8 µg/ml FLC plate (n=18)

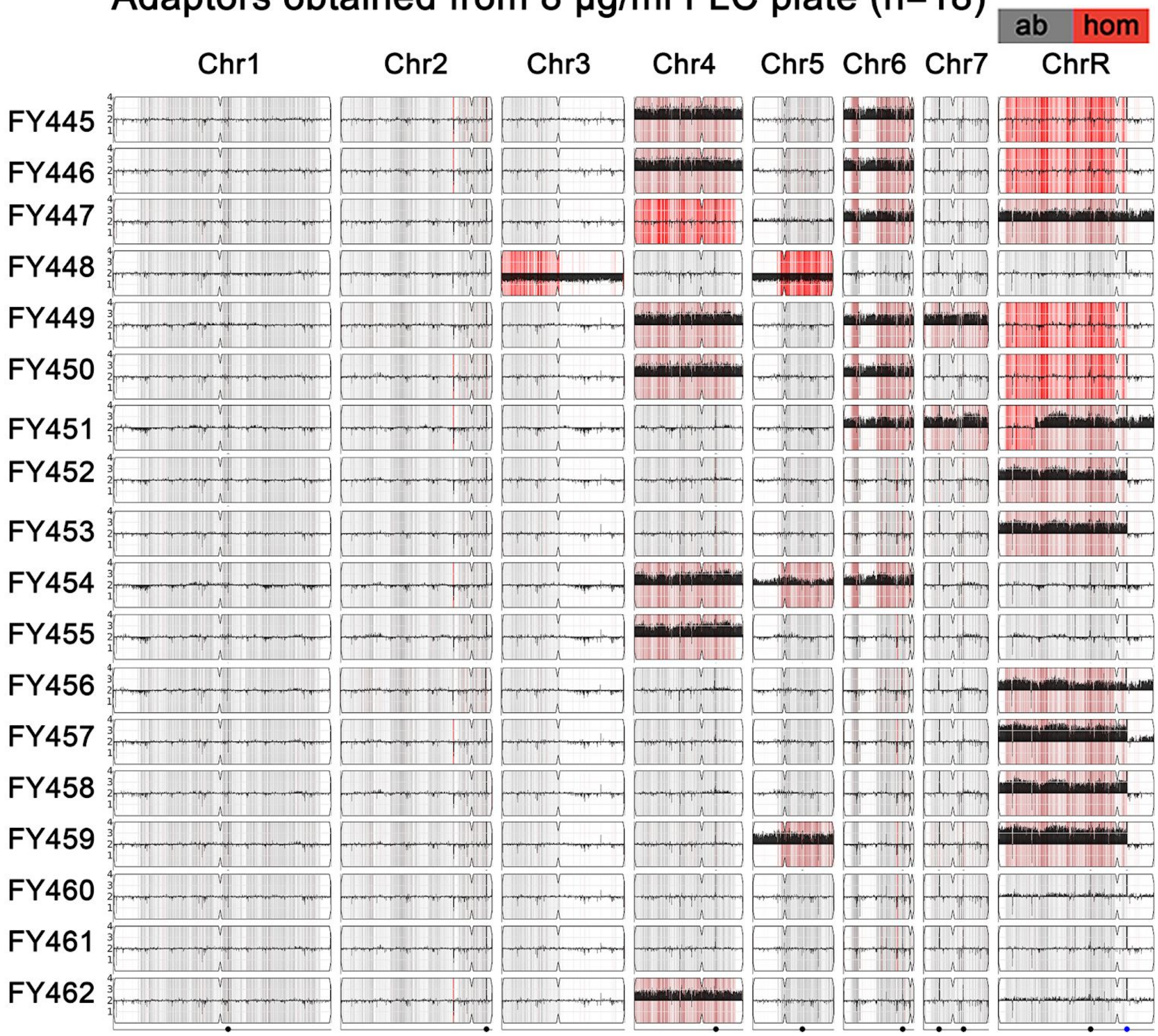

Adaptors obtained from 32 µg/ml FLC plate (n=18)

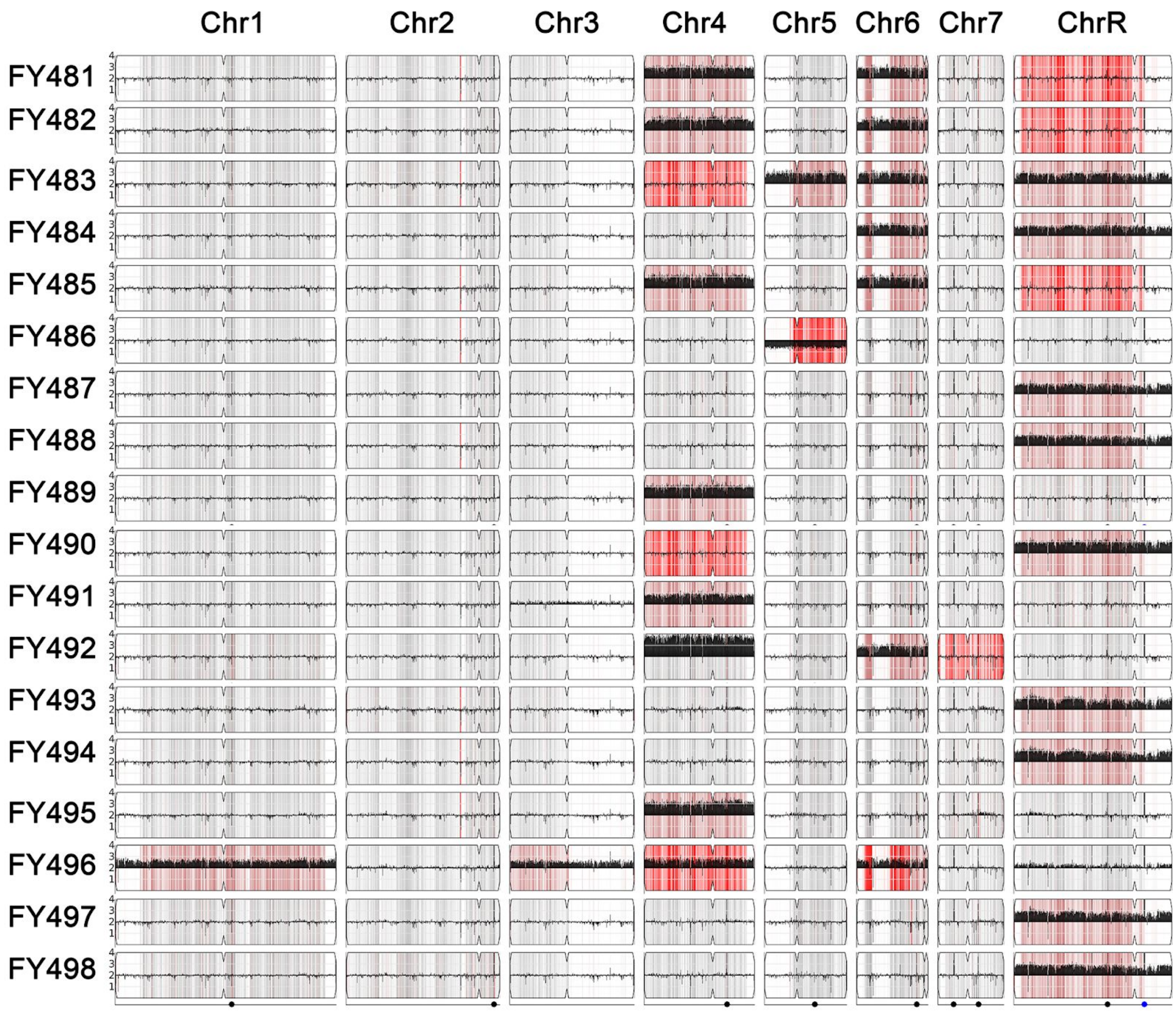

Adaptors obtained from 128 µg/ml FLC plate (n=18)

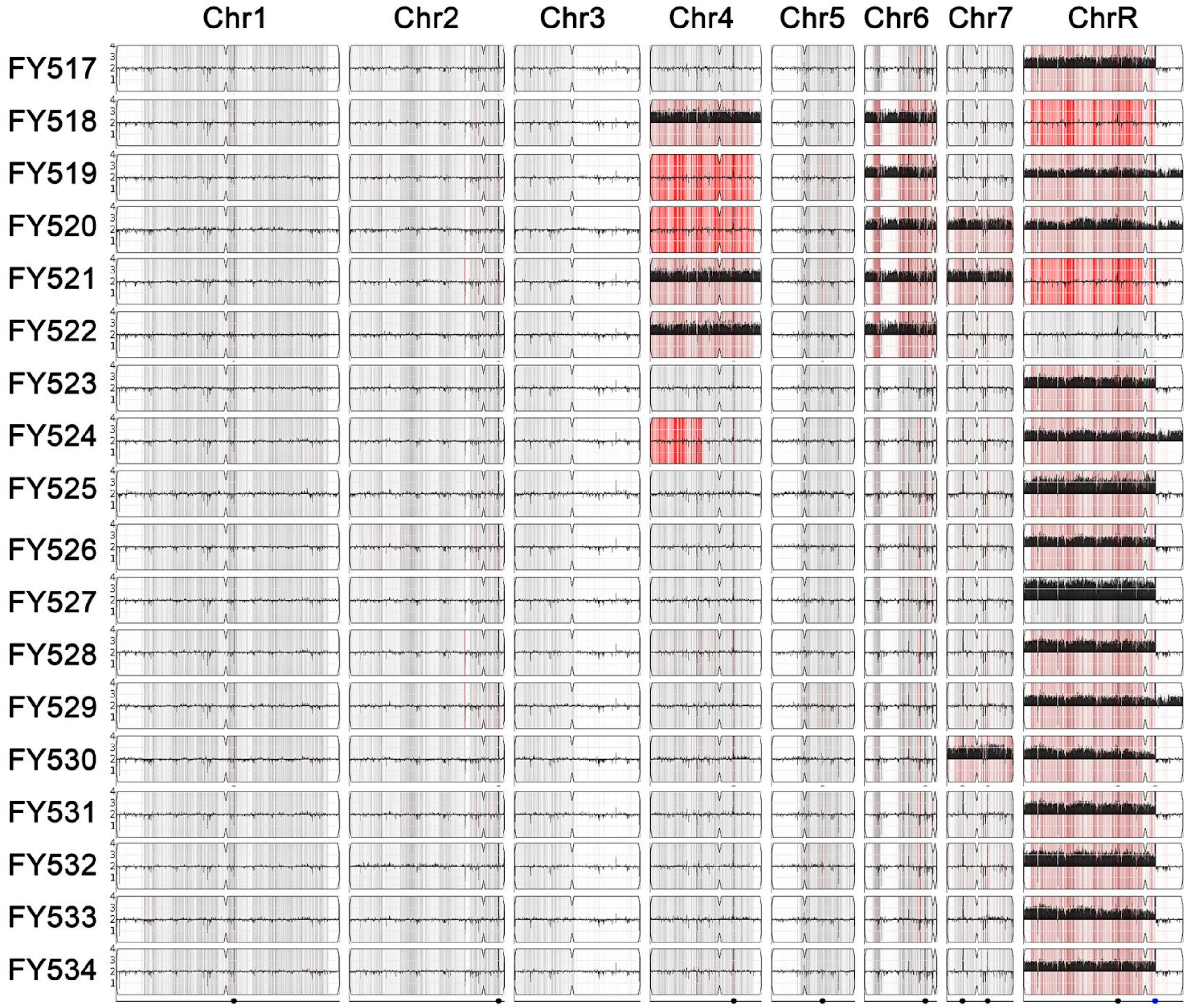

Supplement: FIG S4 [file mbio.00227-23-s0008.pdf]
