# Supplementary material for: Antifungal Tolerance and Resistance Emerge at Distinct Drug Concentrations and Rely upon Different Aneuploid Chromosomes
Source: mBio. 2023 Mar 6;14(2):e00227-23. doi: 10.1128/mbio.00227-23 (PMC10127634; doi:10.1128/mbio.00227-23)
Supplement: TABLE S1 [file mbio.00227-23-s0001.pdf]

New Table S1 CSV

chromosome	Systemic name	Gene Name	CGID	position	Strain	reference allele	alternate allele	mutation	ORF	Allele Frequency	Description
**Chr1**	C1_06340W_A	C1_06340W_A	CAL0000179810	1347766	FY12	G	C	c.2926G>C	Uncharacterized	0.47	(orf19.6277) Ortholog of <i>C. dubliniensis CD36</i> : Cd36_05920, <i>C. parapsilosis CDC317</i> : CPAR2_803860, <i>C. auris B8441</i> : B9J08_000594, <i>Pichia stipitis Pignal</i> : PICST_30324 and <i>Candida guilli
**Chr2**	C2_08380C_A	C2_08380C_A	CAL0000188846	1692880	FY12	ACGA	ACGC	c.1447T>G	Uncharacterized	0.5	(orf19.1434) Ortholog(s) have DNA polymerase binding, protein kinase activator activity, signaling adaptor activity
**Chr1**	C1_02430C_A	C1_02430C_A	CAL0000199029	515797	FY17	T	C	c.1729A>G	Uncharacterized	0.59	(orf19.2930) Predicted translation initiation factor role in translational initiation Spider biofilm repressed
**Chr1**	C1_06340W_A	C1_06340W_A	CAL0000179810	1347766	FY17	G	C	c.2926G>C	Uncharacterized	0.37	(orf19.6277) Ortholog of <i>C. dubliniensis CD36</i> : Cd36_05920, <i>C. parapsilosis CDC317</i> : CPAR2_803860, <i>C. auris B8441</i> : B9J08_000594, <i>Pichia stipitis Pignal</i> : PICST_30324 and <i>Candida guilli
**Chr6**	C6_01500C_A	TRYÊ5.00	CAL0000180184	298883	FY17	C	T	c.2294G>A	Verified	0.55	(orf19.3434) Zn(II)2Cys6 transcription factor regulator of yeast form adherence required for yeast cell adherence to silicone substrate
**ChrR**	CR_09240C_A	CR_09240C_A	CAL0000185958	1967383	FY37	T	C	c.188A>G	Verified	0.46	(orf19.7322) Protein of unknown function S. cerevisae ortholog Ypl225w interacts with ribosomes rat catheter biofilm induced
**Chr1**	C1_06340W_A	C1_06340W_A	CAL0000179810	1347766	FY460	G	C	c.2926G>C	Uncharacterized	0.83	(orf19.6277) Ortholog of <i>C. dubliniensis CD36</i> : Cd36_05920, <i>C. parapsilosis CDC317</i> : CPAR2_803860, <i>C. auris B8441</i> : B9J08_000594, <i>Pichia stipitis Pignal</i> : PICST_30324 and <i>Candida guilli
**Chr1**	C1_06440C_A	C1_06440C_A	CAL0000189377	1374095	FY460	C	A	c.1319G>T	Uncharacterized	0.36	(orf19.6266) Protein of unknown function Spider biofilm induced
**Chr1**	C1_06930W_A	PGA63	CAL0000193385	1477312	FY460	A	G	c.1664A>G	Uncharacterized	0.59	(orf19.6217) Component COPII vesicle coat required for vesicle formation in ER to Golgi transport predicted GPI-anchor flow model and Spider biofilm repressed
**Chr4**	C4_05770C_A	CFL1	CAL0000193490	1274020	FY460	T	G	c.464A>C	Verified	0.36	(orf19.1263) Protein similar to ferric reductase Fre10p possible functional homolog of S. cerevisiae Fre1p (reports differ) transcription is negatively regulated by Sfu1p, copper, amphotericin B, caspofungin induced by ciclopiro
**Chr1**	C1_06340W_A	C1_06340W_A	CAL0000179810	1347766	FY461	G	C	c.2926G>C	Uncharacterized	0.9	(orf19.6277) Ortholog of <i>C. dubliniensis CD36</i> : Cd36_05920, <i>C. parapsilosis CDC317</i> : CPAR2_803860, <i>C. auris B8441</i> : B9J08_000594, <i>Pichia stipitis Pignal</i> : PICST_30324 and <i>Candida guilli
**Chr5**	C5_04370C_A	PGA37	CAL0000201918	965601	FY461	C	T	c.182G>A	Uncharacterized	0.39	(orf19.3923) Putative GPI-anchored protein Hap43-repressed Spider biofilm induced
**Chr6**	C6_04190C_A	C6_04190C_A	CAL0000190667	924299	FY461	C	T	c.463G>A	Uncharacterized	0.6	(orf19.1075) Protein of unknown function Spider biofilm induced
**ChrR**	CR_09520C_A	CR_09520C_A	CAL0000176383	2030975	FY461	T	C	c.790A>G	Uncharacterized	0.89	(orf19.6610) Ortholog(s) have microtubule binding, microtubule plus end polymerase, microtubule plus-end binding activity
