# Supplementary material for: Antifungal Tolerance and Resistance Emerge at Distinct Drug Concentrations and Rely upon Different Aneuploid Chromosomes
Source: mBio. 2023 Mar 6;14(2):e00227-23. doi: 10.1128/mbio.00227-23 (PMC10127634; doi:10.1128/mbio.00227-23)
Supplement: TABLE S2 [file mbio.00227-23-s0002.pdf]

New Table S2 CSV correct

Mutations detected in FY1										
**chromosome**	Systemic name	Gene name	CGID	Position	SNP	Colony Size	Reference Allele	Alternate allele	mutation	ORF
**chr4**	C4_01420W_A	C4_01420W_A	CAL0000190059	297302	C4_01420W_A_297302	Small&Large	C	A	c.2585C>A	Uncharacterized
**chr1**	C1_04090C_A	C1_04090C_A	CAL0000177605	859207	C1_04090C_A_859207	Small	G	A	c.187C>T	Uncharacterized
**chr1**	C1_06340W_A	C1_06340W_A	CAL0000179810	1347766	C1_06340W_A_1347766	Small	G	C	c.2926G>C	Uncharacterized
**chr1**	C1_06340W_A	C1_06340W_A	CAL0000179810	1347780	C1_06340W_A_1347780	Small	T	A	c.2940T>A	Uncharacterized
**chr4**	C4_01450W_A	PWP1	CAL0000198308	307037	C4_01450W_A_307037	Small	A	G	c.1786A>G	Uncharacterized
**chr4**	C4_01450W_A	PWP1	CAL0000198308	307048	C4_01450W_A_307048	Small	A	T	c.1797A>T	Uncharacterized
**chr4**	C4_03350C_A	AIF1	CAL0000191273	702839	C4_03350C_A_702839	Small	G	A	c.1181C>T	Uncharacterized
**chr4**	C4_03970W_A	C4_03970W_A	CAL0000193819	847481	C4_03970W_A_847481	Small	T	C	c.299T>C	Dubious
**chr4**	C4_03970W_A	C4_03970W_A	CAL0000193819	847513	C4_03970W_A_847513	Small	T	C	c.331T>C	Dubious
**chr6**	C6_04190C_A	C6_04190C_A	CAL0000190667	924568	C6_04190C_A_924568	Small	G	A	c.194C>T	Uncharacterized
**chr7**	C7_01130C_A	C7_01130C_A	CAL0000176180	236438	C7_01130C_A_236438	Small	A	T	c.125T>A	Uncharacterized
**chr1**	C1_04140W_A	IFD6	CAL0000201829	867462	C1_04140W_A_867462	Large	G	A	c.83G>A	Verified
**chr1**	C1_06530C_A	C1_06530C_A	CAL0000197778	1390578	C1_06530C_A_1390578	Large	A	T	c.3753T>A	Uncharacterized
**chr1**	C1_08900W_A	C1_08900W_A	CAL0000174928	1935397	C1_08900W_A_1935397	Large	A	G	c.1607A>G	Uncharacterized
**chr2**	C2_07530C_A	C2_07530C_A	CAL0000193545	1536717	C2_07530C_A_1536717	Large	TTC	TTT	c.357G>A	Uncharacterized
**chr2**	C2_08380C_A	C2_08380C_A	CAL0000188846	1692880	C2_08380C_A_1692880	Large	ACGA	ACGC	c.1447T>G	Uncharacterized
**chr4**	C4_01420W_A	C4_01420W_A	CAL0000190059	297310	C4_01420W_A_297310	Large	G	C	c.2593G>C	Uncharacterized
**chr4**	C4_03890W_A	PTP2	CAL0000192372	826712	C4_03890W_A_826712	Large	T	A	c.617T>A	Verified
**chr6**	C6_01020W_A	C6_01020W_A	CAL0000191239	200015	C6_01020W_A_200015	Large	G	A	c.760G>A	Uncharacterized
**Mutations detected in FY13**										
**chr**	Sys	Gene	CGID	pos	SNP	Colony	ref	alt	mutation	ORF
**chr1**	C1_06340W_A	C1_06340W_A	CAL0000179810	1347766	C1_06340W_A_1347766	Small&Large	G	C	c.2926G>C	Uncharacterized
**chr1**	C1_03760C_A	STR2	CAL0000185051	785989	C1_03760C_A_785989	Small	G	T	c.886C>A	Uncharacterized
**chr1**	C1_06340W_A	C1_06340W_A	CAL0000179810	1347780	C1_06340W_A_1347780	Small	T	A	c.2940T>A	Uncharacterized
**chr1**	C1_07780W_A	C1_07780W_A	CAL0000177087	1697491	C1_07780W_A_1697491	Small	A	C	c.866A>C	Uncharacterized
**chr1**	C1_12900W_A	C1_12900W_A	CAL0000196724	2808521	C1_12900W_A_2808521	Small	C	T	c.743C>T	Uncharacterized
**chr2**	C2_06700W_A	AMO2	CAL0000179644	1371636	C2_06700W_A_1371636	Small	T	G	c.16T>G	Verified
**chr2**	C2_06700W_A	AMO2	CAL0000179644	1371639	C2_06700W_A_1371639	Small	T	G	c.19T>G	Verified
**chr2**	C2_08380C_A	C2_08380C_A	CAL0000188846	1692880	C2_08380C_A_1692880	Small	ACGA	ACGC	c.1447T>G	Uncharacterized
**chr3**	C3_05170W_A	WOR2	CAL0000177394	1119011	C3_05170W_A_1119011	Small	G	C	c.287G>C	Verified
**chr4**	C4_01420W_A	C4_01420W_A	CAL0000190059	297302	C4_01420W_A_297302	Small	C	A	c.2585C>A	Uncharacterized
**chr4**	C4_01420W_A	C4_01420W_A	CAL0000190059	297310	C4_01420W_A_297310	Small	G	C	c.2593G>C	Uncharacterized
**chr1**	C1_03710C_A	MSB1	CAL0000199709	767701	C1_03710C_A_767701	Large	T	C	c.3230A>G	Verified
**chr1**	C1_04140W_A	IFD6	CAL0000201829	867462	C1_04140W_A_867462	Large	G	A	c.83G>A	Verified
**chr1**	C1_08900W_A	C1_08900W_A	CAL0000174928	1935447	C1_08900W_A_1935447	Large	A	G	c.1657A>G	Uncharacterized
**chr2**	C2_01140C_A	C2_01140C_A	CAL0000200254	185878	C2_01140C_A_185878	Large	TTC	TTT	c.379G>A	Uncharacterized
**chr2**	C2_08550C_A	MSU1	CAL0000186154	1730264	C2_08550C_A_1730264	Large	GCGG	GCGA	c.1390C>T	Verified
**chr2**	C2_08980C_A	PGA55	CAL0000189083	1825518	C2_08980C_A_1825518	Large	T	A	c.2563A>T	Uncharacterized
**chr4**	C4_01450W_A	PWP1	CAL0000198308	307048	C4_01450W_A_307048	Large	A	T	c.1797A>T	Uncharacterized
**chr4**	C4_06300C_A	PGA42	CAL0000192200	1400954	C4_06300C_A_1400954	Large	T	C	c.427A>G	Uncharacterized
**chr4**	C4_07100C_A	C4_07100C_A	CAL0000181172	1569826	C4_07100C_A_1569826	Large	ACA	ACG	c.4160T>C	Uncharacterized
**chrR**	CR_04950W_A	MED5	CAL0000187701	1071148	CR_04950W_A_1071148	Large	A	T	c.457A>T	Uncharacterized
**Mutations detected in FY42**										
**chr1**	C1_04010C_A	C1_04010C_A	CAL0000175827	843492	C1_04010C_A_843492	Small&Large	CCT	CCA	c.309A>T	Uncharacterized
**chr1**	C1_04890W_A	YBN5	CAL0000180814	1015810	C1_04890W_A_1015810	Small&Large	G	T	c.25G>T	Uncharacterized
**chr1**	C1_06340W_A	C1_06340W_A	CAL0000179810	1347780	C1_06340W_A_1347780	Small&Large	T	A	c.2940T>A	Uncharacterized
**chr2**	C2_04420W_A	IFA4	CAL0000199289	926123	C2_04420W_A_926123	Small&Large	G	C	c.592G>C	Uncharacterized
**chr2**	C2_08380C_A	C2_08380C_A	CAL0000188846	1692880	C2_08380C_A_1692880	Small&Large	ACGA	ACGC	c.1447T>G	Uncharacterized
**chr3**	C3_07830W_A	FBP1	CAL0000200348	1776925	C3_07830W_A_1776925	Small&Large	T	A	c.878T>A	Verified
**chr5**	C5_01590W_A	C5_01590W_A	CAL0000191482	354649	C5_01590W_A_354649	Small&Large	G	A	c.1243G>A	Uncharacterized
**chr1**	C1_06340W_A	C1_06340W_A	CAL0000179810	1347766	C1_06340W_A_1347766	Small	G	C	c.2926G>C	Uncharacterized
**chr3**	C3_03050C_A	PGA18	CAL0000183589	644703	C3_03050C_A_644703	Small	A	G	c.1600T>C	Uncharacterized
**chr6**	C6_01080C_A	C6_01080C_A	CAL0000185118	212274	C6_01080C_A_212274	Small	C	T	c.518G>A	Uncharacterized
**chr7**	C7_02350C_A	C7_02350C_A	CAL0000176532	512066	C7_02350C_A_512066	Small	T	A	c.2139A>T	Uncharacterized
**chr1**	C1_00010W_A	C1_00010W_A	CAL0000182519	4209	C1_00010W_A_4209	Large	A	G	c.151A>G	Dubious
**chr1**	C1_02370C_A	C1_02370C_A	CAL0000194228	495134	C1_02370C_A_495134	Large	G	T	c.1084C>A	Uncharacterized
**chr1**	C1_04140W_A	IFD6	CAL0000201829	867462	C1_04140W_A_867462	Large	G	A	c.83G>A	Verified
**chr1**	C1_12900W_A	C1_12900W_A	CAL0000196724	2808521	C1_12900W_A_2808521	Large	C	T	c.743C>T	Uncharacterized
**chr2**	C2_04790C_A	C2_04790C_A	CAL0000183512	1004968	C2_04790C_A_1004968	Large	T	A	c.544A>T	Uncharacterized
**chr2**	C2_06600W_A	SKY2	CAL0000196384	1343090	C2_06600W_A_1343090	Large	T	A	c.2080T>A	Verified
**chr3**	C3_02410C_A	C3_02410C_A	CAL0000185484	527125	C3_02410C_A_527125	Large	T	C	c.1220A>G	Uncharacterized
**chr4**	C4_07100C_A	C4_07100C_A	CAL0000181172	1569826	C4_07100C_A_1569826	Large	ACA	ACG	c.4160T>C	Uncharacterized
**chr6**	C6_01020W_A	C6_01020W_A	CAL0000191239	200015	C6_01020W_A_200015	Large	G	A	c.760G>A	Uncharacterized
**Mutations detected in FY73**										
**chr**	Sys	Gene	CGID	pos	SNP	Colony	ref	alt	mutation	ORF
**chr1**	C1_06340W_A	C1_06340W_A	CAL0000179810	1347766	C1_06340W_A_1347766	Small&Large	G	C	c.2926G>C	Uncharacterized
**chr1**	C1_06340W_A	C1_06340W_A	CAL0000179810	1347780	C1_06340W_A_1347780	Small&Large	T	A	c.2940T>A	Uncharacterized
**chr4**	C4_05370W_A	PUF3	CAL0000187550	1171192	C4_05370W_A_1171192	Small&Large	C	T	c.170C>T	Verified
**chr1**	C1_04010C_A	C1_04010C_A	CAL0000175827	843482	C1_04010C_A_843482	Small	C	A	c.321G>T	Uncharacterized
**chr1**	C1_12900W_A	C1_12900W_A	CAL0000196724	2808521	C1_12900W_A_2808521	Small	C	T	c.743C>T	Uncharacterized
**chr6**	C6_01570C_A	FGR47	CAL0000190027	322154	C6_01570C_A_322154	Small	C	T	c.2248G>A	Verified
**chr1**	C1_00010W_A	C1_00010W_A	CAL0000182519	4209	C1_00010W_A_4209	Large	A	G	c.151A>G	Dubious
**chr1**	C1_04140W_A	IFD6	CAL0000201829	867462	C1_04140W_A_867462	Large	G	A	c.83G>A	Verified
**chr1**	C1_08900W_A	C1_08900W_A	CAL0000174928	1935397	C1_08900W_A_1935397	Large	A	G	c.1607A>G	Uncharacterized
**chr2**	C2_04790C_A	C2_04790C_A	CAL0000183512	1004968	C2_04790C_A_1004968	Large	T	A	c.544A>T	Uncharacterized
**chr2**	C2_08380C_A	C2_08380C_A	CAL0000188846	1692880	C2_08380C_A_1692880	Large	ACGA	ACGC	c.1447T>G	Uncharacterized
**chr4**	C4_07100C_A	C4_07100C_A	CAL0000181172	1569826	C4_07100C_A_1569826	Large	ACA	ACG	c.4160T>C	Uncharacterized
**chr6**	C6_01020W_A	C6_01020W_A	CAL0000191239	200015	C6_01020W_A_200015	Large	G	A	c.760G>A	Uncharacterized
**chr6**	C6_01020W_A	C6_01020W_A	CAL0000191239	200109	C6_01020W_A_200109	Large	A	G	c.854A>G	Uncharacterized
**chr6**	C6_01030W_A	HAL22	CAL0000200084	201548	C6_01030W_A_201548	Large	G	A	c.1031G>A	Uncharacterized
**chr6**	C6_01080C_A	C6_01080C_A	CAL0000185118	212274	C6_01080C_A_212274	Large	C	T	c.518G>A	Uncharacterized
**chr7**	C7_02850W_A	C7_02850W_A	CAL0000192400	615653	C7_02850W_A_615653	Large	A	G	c.737A>G	Uncharacterized
**chrR**	CR_08100C_A	RIO2	CAL0000193362	1765810	CR_08100C_A_1765810	Large	G	T	c.8C>A	Uncharacterized
**Mutations detected in FY83**										
**chr**	Sys	Gene	CGID	pos	SNP	Colony	ref	alt	mutation	ORF
**chr1**	C1_06340W_A	C1_06340W_A	CAL0000179810	1347766	C1_06340W_A_1347766	Small&Large	G	C	c.2926G>C	Uncharacterized
**chr1**	C1_06340W_A	C1_06340W_A	CAL0000179810	1347780	C1_06340W_A_1347780	Small&Large	T	A	c.2940T>A	Uncharacterized
**chr1**	C1_10950C_A	C1_10950C_A	CAL0000187360	2417395	C1_10950C_A_2417395	Small&Large	C	A	c.816G>T	Uncharacterized
**chr1**	C1_14350W_A	C1_14350W_A	CAL0000174216	3162465	C1_14350W_A_3162465	Small&Large	G	T	c.388G>T	Uncharacterized
**chr6**	C6_01500C_A	TRY 5.00	CAL0000180184	298883	C6_01500C_A_298883	Small&Large	C	T	c.2294G>A	Verified
**chr6**	C6_01570C_A	FGR47	CAL0000190027	322154	C6_01570C_A_322154	Small&Large	C	T	c.2248G>A	Verified
**chrR**	CR_04950W_A	MED5	CAL0000187701	1071148	CR_04950W_A_1071148	Small	A	T	c.457A>T	Uncharacterized
**chr1**	C1_04010C_A	C1_04010C_A	CAL0000175827	843492	C1_04010C_A_843492	Large	CCT	CCA	c.309A>T	Uncharacterized
**chr1**	C1_12810W_A	RIB7	CAL0000179348	2790915	C1_12810W_A_2790915	Large	G	A	c.752G>A	Uncharacterized
**chr1**	C1_12900W_A	C1_12900W_A	CAL0000196724	2808521	C1_12900W_A_2808521	Large	C	T	c.743C>T	Uncharacterized
**chr2**	C2_08380C_A	C2_08380C_A	CAL0000188846	1692880	C2_08380C_A_1692880	Large	ACGA	ACGC	c.1447T>G	Uncharacterized
**chr4**	C4_06300C_A	PGA42	CAL0000192200	1400954	C4_06300C_A_1400954	Large	T	C	c.427A>G	Uncharacterized
**chr4**	C4_07100C_A	C4_07100C_A	CAL0000181172	1569826	C4_07100C_A_1569826	Large	ACA	ACG	c.4160T>C	Uncharacterized
**chr5**	C5_01590W_A	C5_01590W_A	CAL0000191482	354649	C5_01590W_A_354649	Large	G	A	c.1243G>A	Uncharacterized
**Mutations detected in FY87**										
**chr**	Sys	Gene	CGID	pos	SNP	Colony	ref	alt	mutation	ORF
**chr1**	C1_00010W_A	C1_00010W_A	CAL0000182519	4209	C1_00010W_A_4209	Small&Large	A	G	c.151A>G	Dubious
**chr1**	C1_04010C_A	C1_04010C_A	CAL0000175827	843482	C1_04010C_A_843482	Small&Large	C	A	c.321G>T	Uncharacterized
**chr1**	C1_06340W_A	C1_06340W_A	CAL0000179810	1347780	C1_06340W_A_1347780	Small&Large	T	A	c.2940T>A	Uncharacterized
**chr1**	C1_02370C_A	C1_02370C_A	CAL0000194228	495134	C1_02370C_A_495134	Small	G	T	c.1084C>A	Uncharacterized
**chr1**	C1_06340W_A	C1_06340W_A	CAL0000179810	1347766	C1_06340W_A_1347766	Small	G	C	c.2926G>C	Uncharacterized
**chr1**	C1_08900W_A	C1_08900W_A	CAL0000174928	1935447	C1_08900W_A_1935447	Small	A	G	c.1657A>G	Uncharacterized
**chr3**	C3_03050C_A	PGA18	CAL0000183589	644703	C3_03050C_A_644703	Small	A	G	c.1600T>C	Uncharacterized
**chr6**	C6_03700W_A	ALS1	CAL0000199010	793568	C6_03700W_A_793568	Small	T	C	c.2278T>C	Verified
**chr1**	C1_06340W_A	C1_06340W_A	CAL0000179810	1347755	C1_06340W_A_1347755	Large	TTGATGATGATG.	TTGATGATGAT(	c.2926G>C	Uncharacterized
**chr1**	C1_08900W_A	C1_08900W_A	CAL0000174928	1935397	C1_08900W_A_1935397	Large	A	G	c.1607A>G	Uncharacterized
**chr1**	C1_08900W_A	C1_08900W_A	CAL0000174928	1935409	C1_08900W_A_1935409	Large	A	G	c.1619A>G	Uncharacterized
**chr1**	C1_11730W_A	C1_11730W_A	CAL0000201060	2570080	C1_11730W_A_2570080	Large	T	A	c.1395T>A	Uncharacterized
**chr4**	C4_02540W_A	RGT1	CAL0000176948	526667	C4_02540W_A_526667	Large	A	C	c.2788A>C	Verified

Description
(orf19.4643) Ortholog of <i>C. dubliniensis CD36</i> : Cd36_41430, <i>Candida tropicalis NEW ASSEMBLY</i> : CTRG1_00187, <i>Candida tropicalis MYA-3404</i> : CTRG_00187 and <i>Candida albicans WO-1</i> : CAV
(orf19.1041) Ortholog(s) have cyclin-dependent protein serine/threonine kinase activator activity
(orf19.6277) Ortholog of <i>C. dubliniensis CD36</i> : Cd36_05920, <i>C. parapsilosis CDC317</i> : CPAR2_803860, <i>C. auris B8441</i> : B9J08_000594, <i>Pichia stipitis Pignal</i> : PICST_30324 and <i>Candida guilli
(orf19.6277) Ortholog of <i>C. dubliniensis CD36</i> : Cd36_05920, <i>C. parapsilosis CDC317</i> : CPAR2_803860, <i>C. auris B8441</i> : B9J08_000594, <i>Pichia stipitis Pignal</i> : PICST_30324 and <i>Candida guilli
(orf19.4640) Putative rRNA processing protein Hap43-induced repressed in core stress response
(orf19.4640) Putative rRNA processing protein Hap43-induced repressed in core stress response
(orf19.3362) Has domain(s) with predicted flavin adenine dinucleotide binding, oxidoreductase activity
(orf19.1207) Ortholog of <i>C. dubliniensis CD36</i> : Cd36_33200 and <i>Candida albicans WO-1</i> : CAWG_03416
(orf19.1207) Ortholog of <i>C. dubliniensis CD36</i> : Cd36_33200 and <i>Candida albicans WO-1</i> : CAWG_03416
(orf19.1075) Protein of unknown function Spider biofilm induced
(orf19.6897) Protein of unknown function
(orf19.1048) Aldo-keto reductase similar to aryl alcohol dehydrogenases protein increase correlates with MDR1 overexpression (not CDR1 or CDR2) in fluconazole-resistant clinical isolates farnesol regulated possibly essentia
(orf19.6260) Ubiquitin-specific protease cleaves ubiquitin from ubiquitinated proteins Spider biofilm induced
(orf19.4749) Protein of unknown function hyphal-induced expression, regulated by Cyr1, Ras1, Efg1 Hap43-induced gene Spider biofilm induced
(orf19.1874) Ortholog(s) have protein serine/threonine kinase activity
(orf19.1434) Ortholog(s) have DNA polymerase binding, protein kinase activator activity, signaling adaptor activity
(orf19.4643) Ortholog of <i>C. dubliniensis CD36</i> : Cd36_41430, <i>Candida tropicalis NEW ASSEMBLY</i> : CTRG1_00187, <i>Candida tropicalis MYA-3404</i> : CTRG_00187 and <i>Candida albicans WO-1</i> : CAV
(orf19.5045) Predicted protein tyrosine phosphatase involved in regulation of MAP kinase Hog1 activity induced by Mnl1 under weak acid stress rat catheter and Spider biofilm induced
(orf19.104) Protein of unknown function induced by alpha pheromone in SpiderM medium
desc
(orf19.6277) Ortholog of <i>C. dubliniensis CD36</i> : Cd36_05920, <i>C. parapsilosis CDC317</i> : CPAR2_803860, <i>C. auris B8441</i> : B9J08_000594, <i>Pichia stipitis Pignal</i> : PICST_30324 and <i>Candida guilli
(orf19.1033) Ortholog(s) have cystathionine gamma-synthase activity and role in sulfur compound metabolic process, transsulfuration
(orf19.6277) Ortholog of <i>C. dubliniensis CD36</i> : Cd36_05920, <i>C. parapsilosis CDC317</i> : CPAR2_803860, <i>C. auris B8441</i> : B9J08_000594, <i>Pichia stipitis Pignal</i> : PICST_30324 and <i>Candida guilli
(orf19.4713) Ortholog of <i>C. dubliniensis CD36</i> : Cd36_07300, <i>C. parapsilosis CDC317</i> : CPAR2_208300, <i>C. auris B8441</i> : B9J08_001596 and <i>Candida tenuis NRRL Y-1498</i> : CANTEDRAFT_95879
(orf19.4921) Ortholog of <i>C. dubliniensis CD36</i> : Cd36_12030 and <i>Candida albicans WO-1</i> : CAWG_00152
(orf19.3152) Protein similar to A. niger predicted peroxisomal copper amino oxidase mutation confers hypersensitivity to toxic ergosterol analog F-12/CO2 early biofilm induced
(orf19.3152) Protein similar to A. niger predicted peroxisomal copper amino oxidase mutation confers hypersensitivity to toxic ergosterol analog F-12/CO2 early biofilm induced
(orf19.1434) Ortholog(s) have DNA polymerase binding, protein kinase activator activity, signaling adaptor activity
(orf19.5992) Zn(II)2Cys6 transcription factor regulator of white-opaque switching required for maintenance of opaque state Hap43-induced
(orf19.4643) Ortholog of <i>C. dubliniensis CD36</i> : Cd36_41430, <i>Candida tropicalis NEW ASSEMBLY</i> : CTRG1_00187, <i>Candida tropicalis MYA-3404</i> : CTRG_00187 and <i>Candida albicans WO-1</i> : CAV
(orf19.4643) Ortholog of <i>C. dubliniensis CD36</i> : Cd36_41430, <i>Candida tropicalis NEW ASSEMBLY</i> : CTRG1_00187, <i>Candida tropicalis MYA-3404</i> : CTRG_00187 and <i>Candida albicans WO-1</i> : CAV
(orf19.1133) Putative regulator of transcription expression in S. cerevisiae flo8 and flo11 mutants suggests a role in regulation of adhesion factors
(orf19.1048) Aldo-keto reductase similar to aryl alcohol dehydrogenases protein increase correlates with MDR1 overexpression (not CDR1 or CDR2) in fluconazole-resistant clinical isolates farnesol regulated possibly essentia
(orf19.4749) Protein of unknown function hyphal-induced expression, regulated by Cyr1, Ras1, Efg1 Hap43-induced gene Spider biofilm induced
(orf19.2010) Ortholog of <i>C. dubliniensis CD36</i> : Cd36_16030, <i>C. parapsilosis CDC317</i> : CPAR2_213800, <i>Debaryomyces hansenii CBS767</i> : DEHA2A01320g and <i>Pichia stipitis Pignal</i> : PICST_2943
(orf19.3624) Exoribonuclease, along with helicase Suv3p forms a two-component complex (mtEXO) responsible for mitochondrial RNA degradation
(orf19.207) GPI-anchored adhesin-like protein filament induced regulated by Nrg1, Tup1 regulated upon hyphal formation mRNA binds to She3 and is localized to yeast-form buds and hyphal tips induced during chlamydospo
(orf19.4640) Putative rRNA processing protein Hap43-induced repressed in core stress response
(orf19.2907) Putative GPI-anchored adhesin-like protein decreased transcription is observed in azole-resistant strains that overexpress CDR1 and CDR2 or MDR1
(orf19.3098) Predicted RNA-dependent ATPase RNA helicase Hap43-induced gene
(orf19.1808) RNA polymerase II mediator complex subunit transcription positively regulated by Tbf1p
(orf19.4476) Protein with a NADP-dependent oxidoreductase domain transcript induced by ketoconazole rat catheter and Spider biofilm induced
(orf19.754) P-loop ATPase with similarity to human OLA1 and bacterial YchF Spider biofilm repressed
(orf19.6277) Ortholog of <i>C. dubliniensis CD36</i> : Cd36_05920, <i>C. parapsilosis CDC317</i> : CPAR2_803860, <i>C. auris B8441</i> : B9J08_000594, <i>Pichia stipitis Pignal</i> : PICST_30324 and <i>Candida guilli
(orf19.4510) Protein of unknown function oxidative stress-induced via Cap1
(orf19.1434) Ortholog(s) have DNA polymerase binding, protein kinase activator activity, signaling adaptor activity
(orf19.6178) Fructose-1,6-bisphosphatase key gluconeogenesis enzyme regulated by Efg1, Ssn6 induced by phagocytosis effects switch from glycolysis to gluconeogenesis in macrophage rat flow model biofilm induced ove
(orf19.4153) Ortholog(s) have NEDD8 activating enzyme activity and role in protein neddylation
(orf19.6277) Ortholog of <i>C. dubliniensis CD36</i> : Cd36_05920, <i>C. parapsilosis CDC317</i> : CPAR2_803860, <i>C. auris B8441</i> : B9J08_000594, <i>Pichia stipitis Pignal</i> : PICST_30324 and <i>Candida guilli
(orf19.301) Putative GPI-anchored protein regulated by Nrg1, Tup1 rat catheter biofilm repressed
(orf19.114) Ortholog of <i>C. dubliniensis CD36</i> : Cd36_61190, <i>C. parapsilosis CDC317</i> : CPAR2_603230, <i>C. auris B8441</i> : B9J08_001119 and <i>Candida tenuis NRRL Y-1498</i> : cten_CGOB_00054
(orf19.6476) Putative protein with a predicted role in exocytic transport from the Golgi filament induced
(orf19.6115) Dubious open reading frame
(orf19.3694) Ortholog of <i>C. dubliniensis CD36</i> : Cd36_02210, <i>C. parapsilosis CDC317</i> : CPAR2_106350, <i>C. auris B8441</i> : B9J08_000959 and <i>Candida tenuis NRRL Y-1498</i> : CANTEDRAFT_116785
(orf19.1048) Aldo-keto reductase similar to aryl alcohol dehydrogenases protein increase correlates with MDR1 overexpression (not CDR1 or CDR2) in fluconazole-resistant clinical isolates farnesol regulated possibly essentia
(orf19.4921) Ortholog of <i>C. dubliniensis CD36</i> : Cd36_12030 and <i>Candida albicans WO-1</i> : CAWG_00152
(orf19.185) Ortholog(s) have structural constituent of ribosome activity, role in mitochondrial translation, mitochondrial translational initiation and mitochondrial small ribosomal subunit localization
(orf19.35) SR-like protein kinase involved in utilization of dipeptides as nitrogen source rat catheter, flow model, Spider biofilm induced
(orf19.229) Ortholog(s) have ubiquitin-protein transferase activity, role in ubiquitin-dependent protein catabolic process and chromosome, centromeric region, site of double-strand break localization
(orf19.3098) Predicted RNA-dependent ATPase RNA helicase Hap43-induced gene
(orf19.104) Protein of unknown function induced by alpha pheromone in SpiderM medium
desc
(orf19.6277) Ortholog of <i>C. dubliniensis CD36</i> : Cd36_05920, <i>C. parapsilosis CDC317</i> : CPAR2_803860, <i>C. auris B8441</i> : B9J08_000594, <i>Pichia stipitis Pignal</i> : PICST_30324 and <i>Candida guilli
(orf19.6277) Ortholog of <i>C. dubliniensis CD36</i> : Cd36_05920, <i>C. parapsilosis CDC317</i> : CPAR2_803860, <i>C. auris B8441</i> : B9J08_000594, <i>Pichia stipitis Pignal</i> : PICST_30324 and <i>Candida guilli
(orf19.1795) RNA-binding protein involved in regulation of mitochondrial biogenesis
(orf19.4476) Protein with a NADP-dependent oxidoreductase domain transcript induced by ketoconazole rat catheter and Spider biofilm induced
(orf19.4921) Ortholog of <i>C. dubliniensis CD36</i> : Cd36_12030 and <i>Candida albicans WO-1</i> : CAWG_00152
(orf19.3429) Protein lacking an ortholog in S. cerevisiae transposon mutation affects filamentous growth
(orf19.6115) Dubious open reading frame
(orf19.1048) Aldo-keto reductase similar to aryl alcohol dehydrogenases protein increase correlates with MDR1 overexpression (not CDR1 or CDR2) in fluconazole-resistant clinical isolates farnesol regulated possibly essentia
(orf19.4749) Protein of unknown function hyphal-induced expression, regulated by Cyr1, Ras1, Efg1 Hap43-induced gene Spider biofilm induced
(orf19.185) Ortholog(s) have structural constituent of ribosome activity, role in mitochondrial translation, mitochondrial translational initiation and mitochondrial small ribosomal subunit localization
(orf19.1434) Ortholog(s) have DNA polymerase binding, protein kinase activator activity, signaling adaptor activity
(orf19.3098) Predicted RNA-dependent ATPase RNA helicase Hap43-induced gene
(orf19.104) Protein of unknown function induced by alpha pheromone in SpiderM medium
(orf19.104) Protein of unknown function induced by alpha pheromone in SpiderM medium
(orf19.105) Putative phosphoadenosine-5'-phosphate or 3'-phosphoadenosine 5'-phosphosulfate phosphatase possible role in sulfur recycling Hap43-repressed F-12/CO2 biofilm induced
(orf19.114) Ortholog of <i>C. dubliniensis CD36</i> : Cd36_61190, <i>C. parapsilosis CDC317</i> : CPAR2_603230, <i>C. auris B8441</i> : B9J08_001119 and <i>Candida tenuis NRRL Y-1498</i> : cten_CGOB_00054
(orf19.5177) Ortholog of <i>Candida albicans WO-1</i> : CAWG_05627
(orf19.6369) Putative serine kinase with a predicted role in the processing of the 20S pre-rRNA into mature 18S rRNA null mutants are hypersensitive to caspofungin
desc
(orf19.6277) Ortholog of <i>C. dubliniensis CD36</i> : Cd36_05920, <i>C. parapsilosis CDC317</i> : CPAR2_803860, <i>C. auris B8441</i> : B9J08_000594, <i>Pichia stipitis Pignal</i> : PICST_30324 and <i>Candida guilli
(orf19.6277) Ortholog of <i>C. dubliniensis CD36</i> : Cd36_05920, <i>C. parapsilosis CDC317</i> : CPAR2_803860, <i>C. auris B8441</i> : B9J08_000594, <i>Pichia stipitis Pignal</i> : PICST_30324 and <i>Candida guilli
(orf19.2320) Putative serine/threonine-protein kinase possibly an essential gene, disruptants not obtained by UAU1 method
(orf19.7250) Protein of unknown function rat catheter and Spider biofilm induced
(orf19.3434) Zn(II)2Cys6 transcription factor regulator of yeast form adherence required for yeast cell adherence to silicone substrate
(orf19.3429) Protein lacking an ortholog in S. cerevisiae transposon mutation affects filamentous growth
(orf19.1808) RNA polymerase II mediator complex subunit transcription positively regulated by Tbf1p
(orf19.4476) Protein with a NADP-dependent oxidoreductase domain transcript induced by ketoconazole rat catheter and Spider biofilm induced
(orf19.6341) Ortholog(s) have 5-amino-6-(5-phosphoribosylamino)uracil reductase activity and role in riboflavin biosynthetic process
(orf19.4921) Ortholog of <i>C. dubliniensis CD36</i> : Cd36_12030 and <i>Candida albicans WO-1</i> : CAWG_00152
(orf19.1434) Ortholog(s) have DNA polymerase binding, protein kinase activator activity, signaling adaptor activity
(orf19.2907) Putative GPI-anchored adhesin-like protein decreased transcription is observed in azole-resistant strains that overexpress CDR1 and CDR2 or MDR1
(orf19.3098) Predicted RNA-dependent ATPase RNA helicase Hap43-induced gene
(orf19.4153) Ortholog(s) have NEDD8 activating enzyme activity and role in protein neddylation
desc
(orf19.6115) Dubious open reading frame
(orf19.4476) Protein with a NADP-dependent oxidoreductase domain transcript induced by ketoconazole rat catheter and Spider biofilm induced
(orf19.6277) Ortholog of <i>C. dubliniensis CD36</i> : Cd36_05920, <i>C. parapsilosis CDC317</i> : CPAR2_803860, <i>C. auris B8441</i> : B9J08_000594, <i>Pichia stipitis Pignal</i> : PICST_30324 and <i>Candida guilli
(orf19.3694) Ortholog of <i>C. dubliniensis CD36</i> : Cd36_02210, <i>C. parapsilosis CDC317</i> : CPAR2_106350, <i>C. auris B8441</i> : B9J08_000959 and <i>Candida tenuis NRRL Y-1498</i> : CANTEDRAFT_116785
(orf19.6277) Ortholog of <i>C. dubliniensis CD36</i> : Cd36_05920, <i>C. parapsilosis CDC317</i> : CPAR2_803860, <i>C. auris B8441</i> : B9J08_000594, <i>Pichia stipitis Pignal</i> : PICST_30324 and <i>Candida guilli
(orf19.4749) Protein of unknown function hyphal-induced expression, regulated by Cyr1, Ras1, Efg1 Hap43-induced gene Spider biofilm induced
(orf19.301) Putative GPI-anchored protein regulated by Nrg1, Tup1 rat catheter biofilm repressed
(orf19.5741) Cell-surface adhesin adhesion, virulence, immunoprotective roles band at hyphal base Rfg1, Ssk1, Spider biofilm induced flow model biofilm repressed CAI-4 strain background effects promoter bound Bcr1, Tec
(orf19.6277) Ortholog of <i>C. dubliniensis CD36</i> : Cd36_05920, <i>C. parapsilosis CDC317</i> : CPAR2_803860, <i>C. auris B8441</i> : B9J08_000594, <i>Pichia stipitis Pignal</i> : PICST_30324 and <i>Candida guilli
(orf19.4749) Protein of unknown function hyphal-induced expression, regulated by Cyr1, Ras1, Efg1 Hap43-induced gene Spider biofilm induced
(orf19.4749) Protein of unknown function hyphal-induced expression, regulated by Cyr1, Ras1, Efg1 Hap43-induced gene Spider biofilm induced
(orf19.1144) Protein with SEL-1 like protein domain early-stage flow model biofilm induced
(orf19.2747) Zn(II)2Cys6 transcription factor transcriptional repressor involved in the regulation of glucose transporter genes ortholog of S. cerevisiae Rgt1 mutants display decreased colonization of mouse kidneys
