# Supplementary material for: Antifungal Tolerance and Resistance Emerge at Distinct Drug Concentrations and Rely upon Different Aneuploid Chromosomes
Source: mBio. 2023 Mar 6;14(2):e00227-23. doi: 10.1128/mbio.00227-23 (PMC10127634; doi:10.1128/mbio.00227-23)
Supplement: FIG S6 [file mbio.00227-23-s0010.pdf]

Adaptors evolved in 2 μg/ml FLC for 1 day

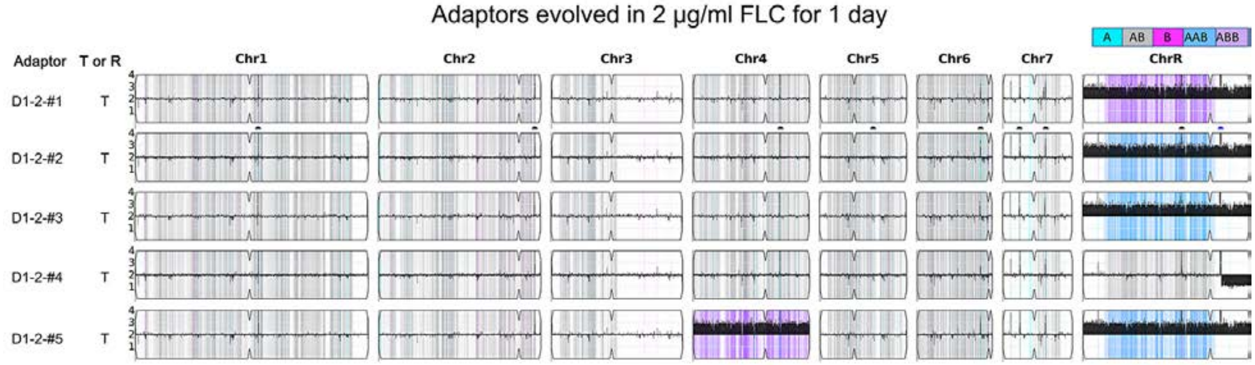

Adaptors evolved in 128 μg/ml FLC for 1 day

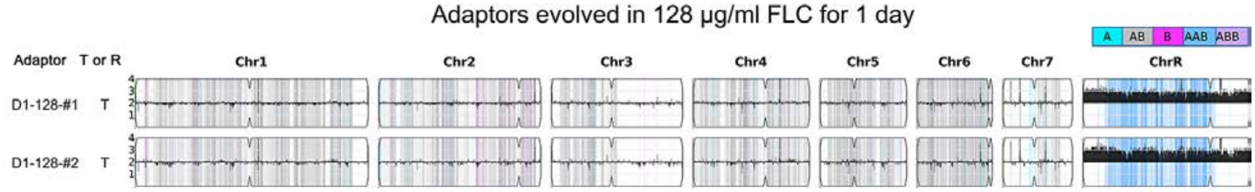

Adaptors evolved in 1 μg/ml FLC for 5 days

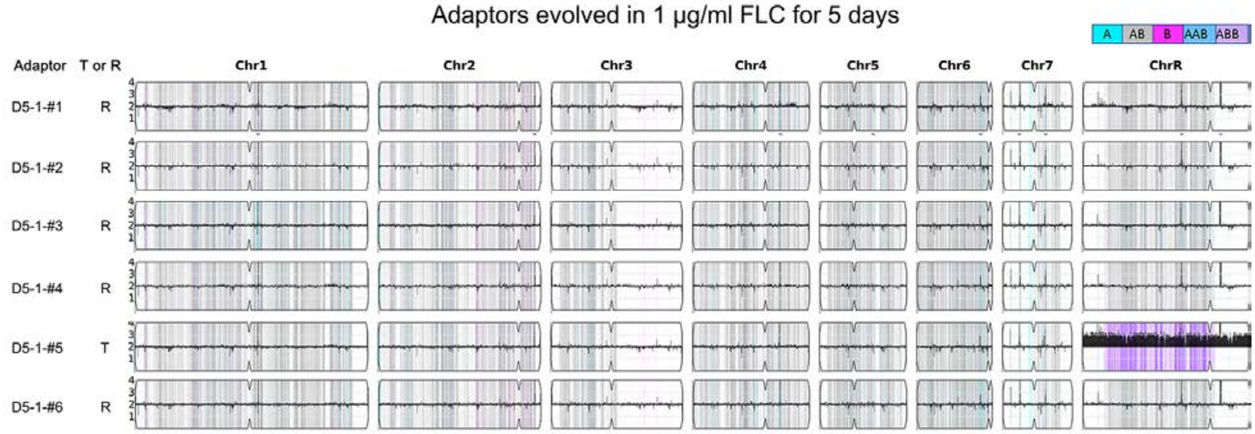

Adaptors evolved in 0.25 μg/ml FLC for 10 days

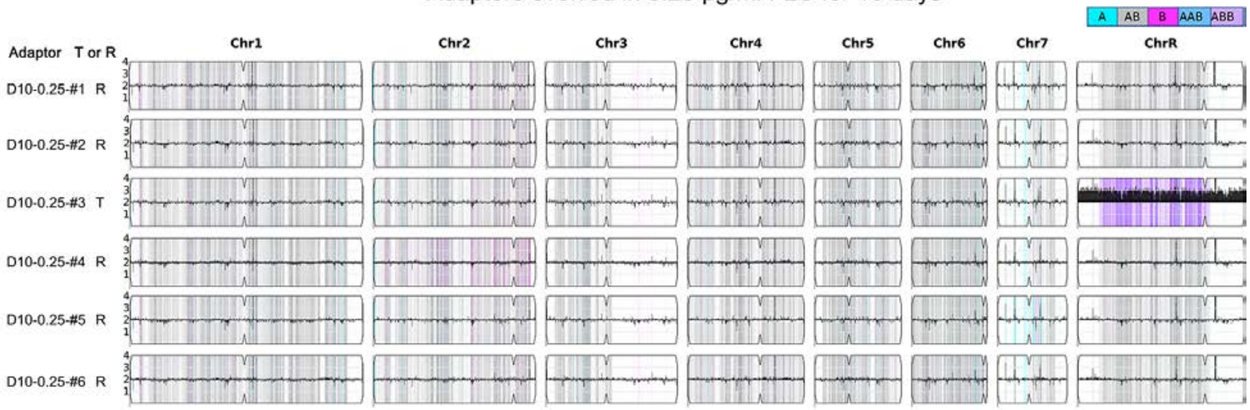

Adaptors evolved in 0.5 μg/ml FLC for 10 days

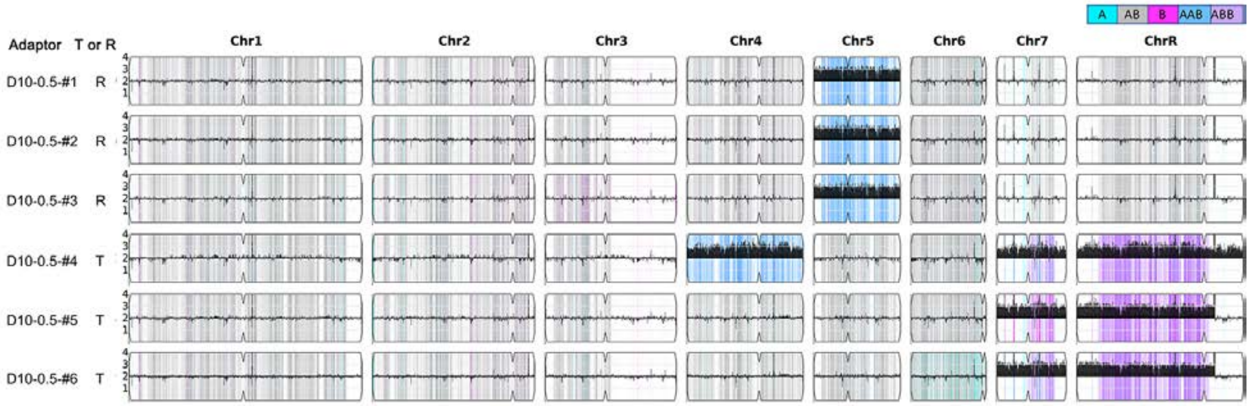

Adaptors evolved in 1 μg/ml FLC for 10 days

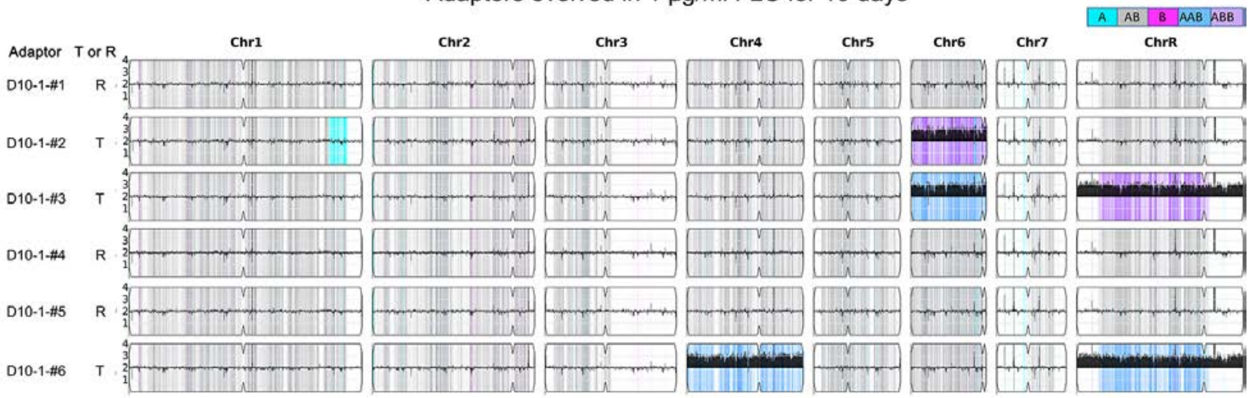

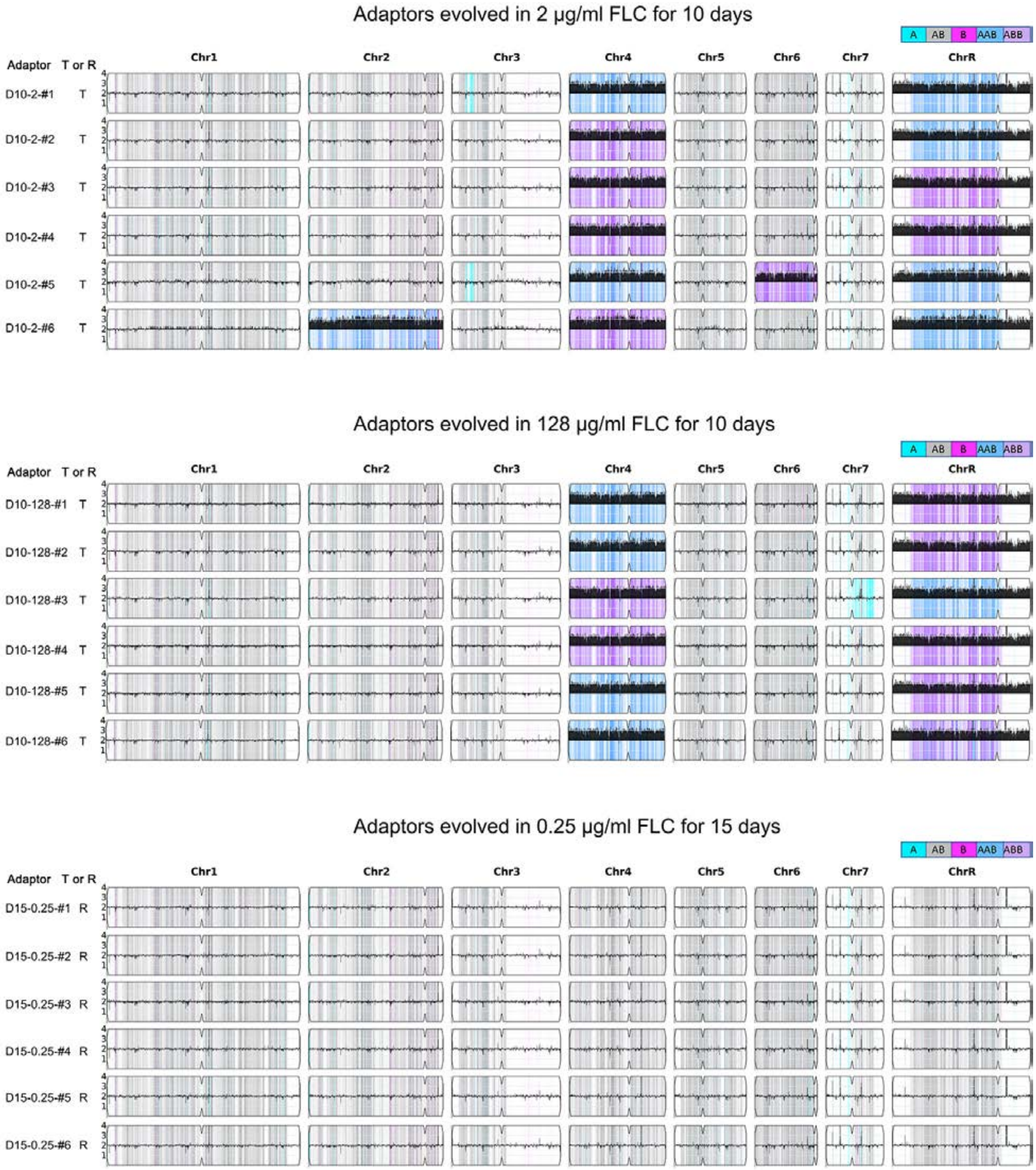
Adaptors evolved in 2 µg/ml FLC for 10 days
A AB B AAB ABB
Adaptor T or R
Chr1 Chr2 Chr3 Chr4 Chr5 Chr6 Chr7 ChrR
D10-2-#1 T
D10-2-#2 T
D10-2-#3 T
D10-2-#4 T
D10-2-#5 T
D10-2-#6 T
Adaptors evolved in 128 µg/ml FLC for 10 days
A AB B AAB ABB
Adaptor T or R
Chr1 Chr2 Chr3 Chr4 Chr5 Chr6 Chr7 ChrR
D10-128-#1 T
D10-128-#2 T
D10-128-#3 T
D10-128-#4 T
D10-128-#5 T
D10-128-#6 T
Adaptors evolved in 0.25 µg/ml FLC for 15 days
A AB B AAB ABB
Adaptor T or R
Chr1 Chr2 Chr3 Chr4 Chr5 Chr6 Chr7 ChrR
D15-0.25-#1 R
D15-0.25-#2 R
D15-0.25-#3 R
D15-0.25-#4 R
D15-0.25-#5 R
D15-0.25-#6 R

Adaptors evolved in 0.5 μg/ml FLC for 15 days

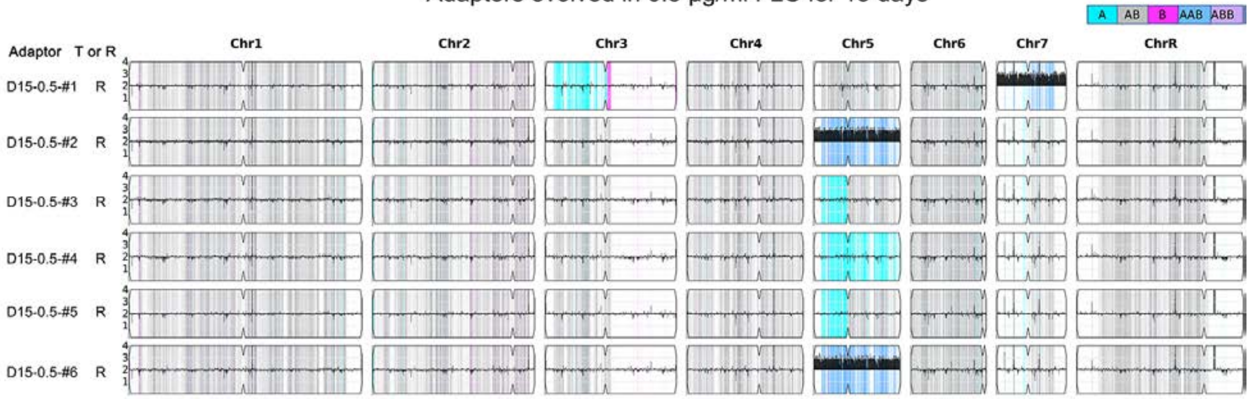

Adaptors evolved in 1 μg/ml FLC for 15 days

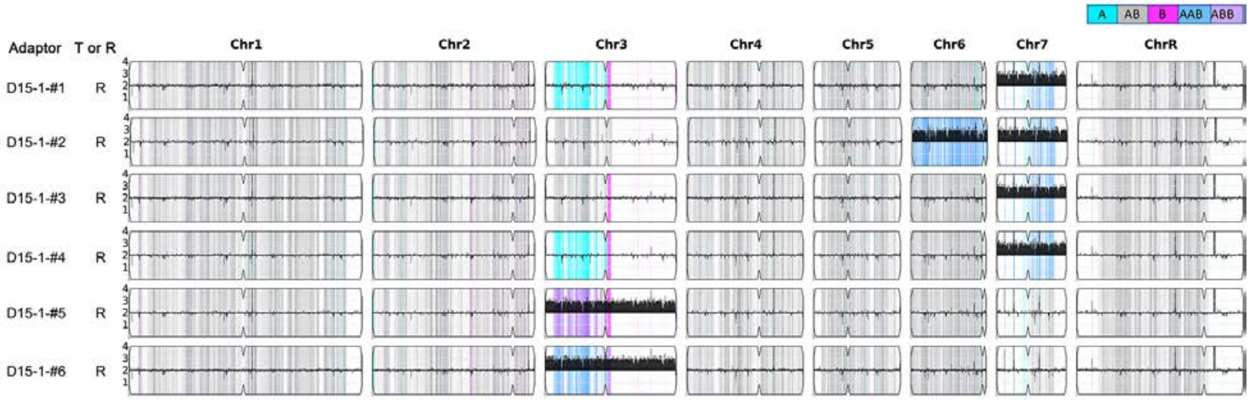

Adaptors evolved in 2 μg/ml FLC for 15 days

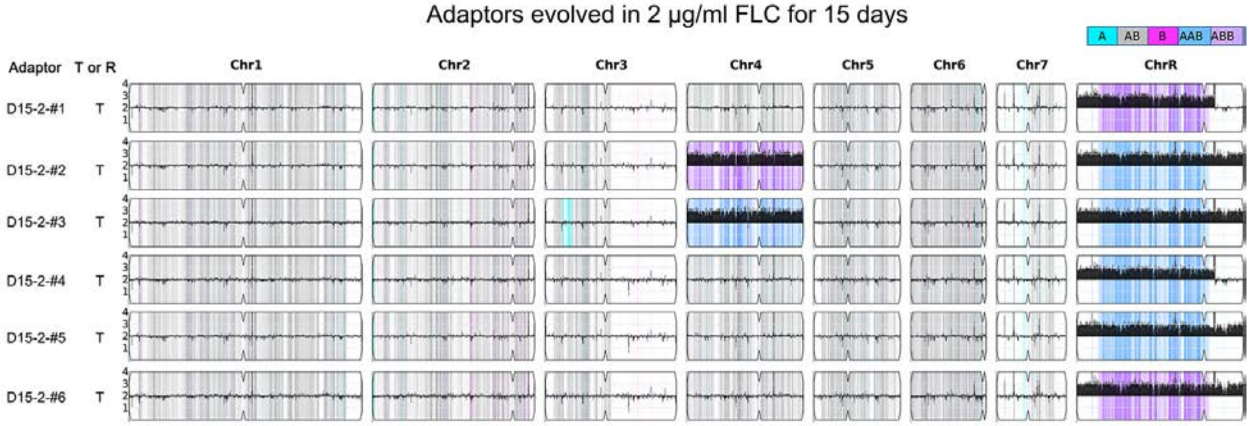

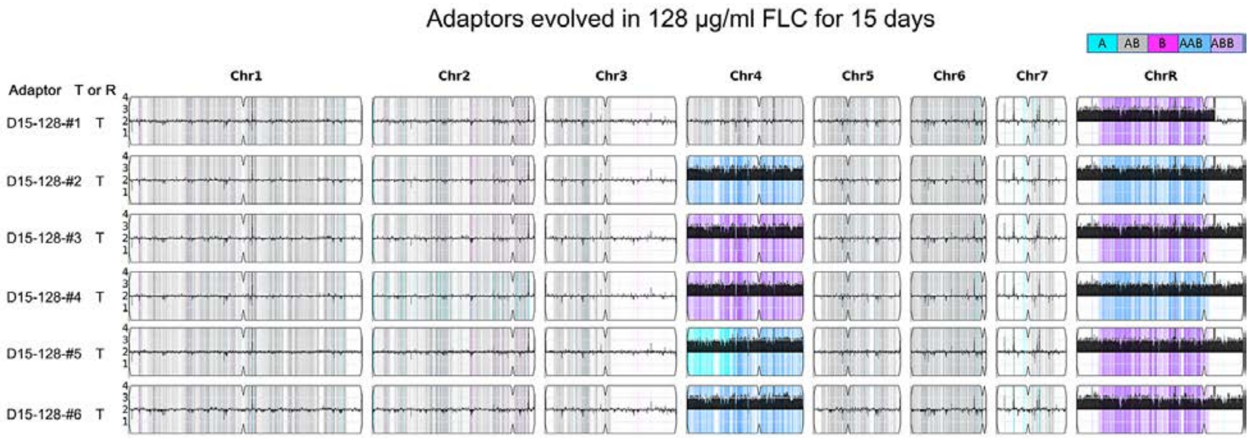
Adaptors evolved in 128 μg/ml FLC for 15 days
A
AB
B
AAB
ABB
Chr1
Chr2
Chr3
Chr4
Chr5
Chr6
Chr7
ChrR
Adaptor T or R
D15-128-#1 T
D15-128-#2 T
D15-128-#3 T
D15-128-#4 T
D15-128-#5 T
D15-128-#6 T
