# Supplementary material for: Antifungal Tolerance and Resistance Emerge at Distinct Drug Concentrations and Rely upon Different Aneuploid Chromosomes
Source: mBio. 2023 Mar 6;14(2):e00227-23. doi: 10.1128/mbio.00227-23 (PMC10127634; doi:10.1128/mbio.00227-23)
Supplement: TABLE S3 [file mbio.00227-23-s0003.pdf]

Table S3

Tolerance-specific mutations												
**SNP**	chromosome	Systemic name	Gene name	CGID	position	Adaptor	referen	alternat	mutation	Allele Frequency	Repetitive	ORF
**C1_02430C_A_515795**	chr1	C1_02430C_A	C1_02430C_A	CAL0000199029	515795	SY64	AGT	AGC	c.1729A>G	0.38	No	Uncharacterized
**C1_03760C_A_785989**	chr1	C1_03760C_A	STR2	CAL0000185051	785989	SY41	G	T	c.886C>A	0.18	No	Uncharacterized
**C1_03860C_A_810301**	chr1	C1_03860C_A	BNI4	CAL0000191087	810301	SY31	G	T	c.1133C>A	0.53	No	Verified
**C1_03920C_A_829766**	chr1	C1_03920C_A	C1_03920C_A	CAL0000174768	829766	SY33	T	A	c.275A>T	0.2	Yes	Uncharacterized
**C1_03960C_A_836807**	chr1	C1_03960C_A	C1_03960C_A	CAL0000177404	836807	SY31	G	T	c.32C>A	0.5	No	Uncharacterized
**C1_04010C_A_843482**	chr1	C1_04010C_A	C1_04010C_A	CAL0000175827	843482	SY38	C	A	c.321G>T	0.25	Yes	Uncharacterized
**C1_04010C_A_843492**	chr1	C1_04010C_A	C1_04010C_A	CAL0000175827	843492	SY41, SY42, SY43	CCT	CCA	c.309A>T	0.28/0.35/0.34	Yes	Uncharacterized
**C1_04140W_A_867462**	chr1	C1_04140W_A	IFD6	CAL0000201829	867462	SY6	G	A	c.83G>A	0.99	No	Verified
**C1_06340W_A_1347780**	chr1	C1_06340W_A	C1_06340W_A	CAL0000179810	1347780	SY38, SY39, SY40, SY41, SY43	T	A	c.2940T>A	0.41/0.42/0.37/0.43/0.37	Yes	Uncharacterized
**C1_07520C_A_1629027**	chr1	C1_07520C_A	IST2	CAL0000183382	1629027	SY37	A	G	c.37T>C	0.45	Yes	Uncharacterized
**C1_07680W_A_1668827**	chr1	C1_07680W_A	HAP2	CAL0000184546	1668827	SY36, SY37	G	A	c.707G>A	0.21/0.17	Yes	Verified
**C1_08900W_A_1935397**	chr1	C1_08900W_A	C1_08900W_A	CAL0000174928	1935397	SY40	A	G	c.1607A>G	0.35	Yes	Uncharacterized
**C1_08900W_A_1935409**	chr1	C1_08900W_A	C1_08900W_A	CAL0000174928	1935409	SY40	A	G	c.1619A>G	0.16	Yes	Uncharacterized
**C1_11590W_A_2542582**	chr1	C1_11590W_A	PLD1	CAL0000189670	2542582	SY35	TG	TA	c.409G>A	0.47	No	Verified
**C1_12810W_A_2790915**	chr1	C1_12810W_A	RIB7	CAL0000179348	2790915	SY36	G	A	c.752G>A	0.16	No	Uncharacterized
**C1_12860C_A_2801568**	chr1	C1_12860C_A	C1_12860C_A	CAL0000178796	2801568	SY38	G	A	c.653C>T	0.2	No	Uncharacterized
**C1_12900W_A_2808521**	chr1	C1_12900W_A	C1_12900W_A	CAL0000196724	2808521	SY38, SY42, SY43	C	T	c.743C>T	0.18/0.24/0.14	Yes	Uncharacterized
**C2_01140C_A_185878**	chr2	C2_01140C_A	C2_01140C_A	CAL0000200254	185878	SY43	TTC	TTT	c.379G>A	0.66	Yes	Uncharacterized
**C2_01140C_A_185879**	chr2	C2_01140C_A	C2_01140C_A	CAL0000200254	185879	SY35	TC	TT	c.379G>A	0.54	Yes	Uncharacterized
**C2_04420W_A_927172**	chr2	C2_04420W_A	IFA4	CAL0000199289	927172	SY42, SY43	C	G	c.1641C>G	0.18/0.15	No	Uncharacterized
**C2_04930C_A_1028968**	chr2	C2_04930C_A	CRK1	CAL0000191033	1028968	SY73	G	T	c.587C>A	0.44	No	Verified
**C2_06470W_A_1317276**	chr2	C2_06470W_A	RTA2	CAL0000175352	1317276	SY66	C	T	c.190C>T	0.48	No	Verified
**C2_06740W_A_1384057**	chr2	C2_06740W_A	C2_06740W_A	CAL0000180208	1384057	SY40	TA	TG	c.880A>G	0.54	No	Uncharacterized
**C2_07530C_A_1536717**	chr2	C2_07530C_A	C2_07530C_A	CAL0000193545	1536717	SY32, SY33, SY36	TTC	TTT	c.357G>A	0.41/0.53/0.53	Yes	Uncharacterized
**C3_00670C_A_113814**	chr3	C3_00670C_A	FKH2	CAL0000195461	113814	SY1	T	C	c.1487A>G	0.45	No	Verified
**C3_05210C_A_1142405**	chr3	C3_05210C_A	DYN1	CAL0000190089	1142405	SY65, SY66, SY67	C	A	c.1367G>T	0.44/0.45/0.56	No	Verified
**C4_07100C_A_1569826**	chr4	C4_07100C_A	C4_07100C_A	CAL0000181172	1569826	SY65, SY66, SY67	ACA	ACG	c.4160T>C	0.48/0.51/0.52	No	Uncharacterized
**CR_01030W_A_233069**	chrR	CR_01030W_A	DAL81	CAL0000195779	233069	SY73	G	A	c.1310G>A	0.38	No	Verified
**CR_04260W_A_954009**	chrR	CR_04260W_A	SNP3	CAL0000178800	954009	SY72	G	A	c.227G>A	0.39	No	Uncharacterized
**CR_09930W_A_2126230**	chrR	CR_09930W_A	CR_09930W_A	CAL0000179961	2126230	SY64	G	T	c.906G>T	0.42	No	Uncharacterized
**Resistance-specific mutations**												
**SNP**	chromosome	Systemic name	Gene name	CGID	position	Adaptor	referen	alternat	mutation	Allele Frequency	Repetitive	ORF
**C1_04740W_A_985545**	chr1	C1_04740W_A	LPG20	CAL0000174538	985545	SY11	G	T	c.817G>T	0.56	No	Uncharacterized
**C1_06530C_A_1390578**	chr1	C1_06530C_A	C1_06530C_A	CAL0000197778	1390578	SY15	A	T	c.3753T>A	0.91	No	Uncharacterized
**C1_13010W_A_2834971**	chr1	C1_13010W_A	C1_13010W_A	CAL0000201653	2834971	SY57	G	A	c.3077G>A	0.54	No	Uncharacterized
**C3_01670W_A_361126**	chr3	C3_01670W_A	C3_01670W_A	CAL0000180987	361126	SY45	A	T	c.590A>T	0.36	No	Uncharacterized
**C3_02220W_A_480264**	chr3	C3_02220W_A	CAP1	CAL0000176112	480264	SY44, SY45, SY46, SY47, SY48, SY49	C	G	c.1364C>G	0.52/0.46/0.50/0.50/0.42/0.46	No	Verified
**C3_06780C_A_1546024**	chr3	C3_06780C_A	SLF1	CAL0000195579	1546024	SY13	T	C	c.13A>G	0.51	No	Uncharacterized
**CR_06350C_A_1346259**	chrR	CR_06350C_A	CR_06350C_A	CAL0000181186	1346259	SY60	T	A	c.89A>T	0.39	No	Dubious
**CR_06350C_A_1346331**	chrR	CR_06350C_A	CR_06350C_A	CAL0000181186	1346331	SY15, SY17	C	T	c.17G>A	0.15/0.15	No	Dubious
**Mutions found in both tolerant and resistant adaptors**												
**SNP**	chromosome	Systemic name	Gene name	CGID	position	Adaptor	referen	alternat	mutation	Allele Frequency	Repetitive	ORF
**C2_08380C_A_1692880**	chr2	C2_08380C_A	C2_08380C_A	CAL0000188846	1692880	SY68, SY69, SY70, SY72, SY73	ACGA	ACGC	c.1447T>G	0.58/0.44/0.55/0.47/0.61	No	Uncharacterized
**C1_02370C_A_495134**	chr1	C1_02370C_A	C1_02370C_A	CAL0000194228	495134	SY69, SY71, SY73	G	T	c.1084C>A	0.41/0.37/0.5	No	Uncharacterized

Description
(orf19.2930) Predicted translation initiation factor role in translational initiation Spider biofilm repressed
(orf19.1033) Ortholog(s) have cystathionine gamma-synthase activity and role in sulfur compound metabolic process, transsulfuration
(orf19.4457) Protein required for wild-type cell wall chitin distribution, morphology, hyphal growth not essential similar to S. cerevisiae Bni4p (targeting subunit for Glc7p phosphatase, involved in bud-neck localization of chitir
(orf19.4466) Ortholog(s) have palmitoyltransferase activity, protein-cysteine S-palmitoyltransferase activity and role in protein palmitoylation, protein targeting to membrane
(orf19.4471) Ortholog(s) have role in TOR signaling, re-entry into mitotic cell cycle after pheromone arrest and endoplasmic reticulum, endoplasmic reticulum membrane, endoplasmic reticulum-Golgi intermediate compartmer
(orf19.4476) Protein with a NADP-dependent oxidoreductase domain transcript induced by ketoconazole rat catheter and Spider biofilm induced
(orf19.4476) Protein with a NADP-dependent oxidoreductase domain transcript induced by ketoconazole rat catheter and Spider biofilm induced
(orf19.1048) Aldo-keto reductase similar to aryl alcohol dehydrogenases protein increase correlates with MDR1 overexpression (not CDR1 or CDR2) in fluconazole-resistant clinical isolates farnesol regulated possibly essentia
(orf19.6277) Ortholog of <i>C. dubliniensis CD36</i> : Cd36_05920, <i>C. parapsilosis CDC317</i> : CPAR2_803860, <i>C. auris B8441</i> : B9J08_000594, <i>Pichia stipitis Pignal</i> : PICST_30324 and <i>Candida guilli
(orf19.2792) Ortholog(s) have lipid binding activity and role in endoplasmic reticulum membrane organization, protein localization to plasma membrane, regulation of phosphatidylinositol dephosphorylation
(orf19.1228) CCAAT-binding transcription factor regulates low-iron induction of FRP1 in these conditions CBF comprises Hap43 and probably Hap2 and Hap3 possibly essential, disruptants not obtained by UAU1 method Ca
(orf19.4749) Protein of unknown function hyphal-induced expression, regulated by Cyr1, Ras1, Efg1 Hap43-induced gene Spider biofilm induced
(orf19.4749) Protein of unknown function hyphal-induced expression, regulated by Cyr1, Ras1, Efg1 Hap43-induced gene Spider biofilm induced
(orf19.1161) Phospholipase D1 required for phosphatidic acid and for most diacylglycerol production required for wild-type mouse virulence, but not rat oral virulence mutant defect in hyphal growth on solid substrates simila
(orf19.6341) Ortholog(s) have 5-amino-6-(5-phosphoribosylamino)uracil reductase activity and role in riboflavin biosynthetic process
(orf19.4916) Protein of unknown function induced by alpha pheromone in SpiderM medium
(orf19.4921) Ortholog of <i>C. dubliniensis CD36</i> : Cd36_12030 and <i>Candida albicans WO-1</i> : CAWG_00152
(orf19.2010) Ortholog of <i>C. dubliniensis CD36</i> : Cd36_16030, <i>C. parapsilosis CDC317</i> : CPAR2_213800, <i>Debaryomyces hansenii CBS767</i> : DEHA2A01320g and <i>Pichia stipitis Pignal</i> : PICST_2943
(orf19.2010) Ortholog of <i>C. dubliniensis CD36</i> : Cd36_16030, <i>C. parapsilosis CDC317</i> : CPAR2_213800, <i>Debaryomyces hansenii CBS767</i> : DEHA2A01320g and <i>Pichia stipitis Pignal</i> : PICST_2943
(orf19.4510) Protein of unknown function oxidative stress-induced via Cap1
(orf19.3523) Protein kinase of the Cdc2 subfamily involved in hyphal development, virulence promotes hyphal development independently of Cph1 and Efg1 functionally complements pheromone hypersensitivity of S. cerevis
(orf19.24) Flippase involved in sphingolipid long chain base release mediates calcineurin-dependent ER stress response and resistance to azoles Plc1p, Ca2+, calcineurin-regulated
(orf19.3148) Protein of unknown function possibly an essential gene, disruptants not obtained by UAU1 method
(orf19.1874) Ortholog(s) have protein serine/threonine kinase activity
(orf19.5389) Forkhead transcription factor morphogenesis regulator required for wild-type hyphal transcription, cell separation, and for virulence in cell culture mutant lacks true hyphae, is constitutively pseudohyphal upregula
(orf19.5999) Dynein heavy chain motor protein that moves to microtubule minus end required for yeast cell separation, spindle positioning, nuclear migration, hyphal growth regulated by Mig1, Hap43 flow model and rat cathe
(orf19.3098) Predicted RNA-dependent ATPase RNA helicase Hap43-induced gene
(orf19.3252) Zn(II)2Cys6 transcription factor ortholog of S. cerevisiae Dal81, involved in the regulation of nitrogen-degradation genes required for yeast cell adherence to silicone substrate Spider biofilm induced
(orf19.514) Putative U6 snRNA-associated protein transcript regulated by Mig1
(orf19.7567) Protein of unknown function induced by alpha pheromone in SpiderM medium
Description
(orf19.771) Aldo-keto reductase family protein similar to aryl alcohol dehydrogenases osmotic stress-induced, correlates with overexpression of MDR1 in fluconazole-resistant isolate stationary phase enriched protein
(orf19.6260) Ubiquitin-specific protease cleaves ubiquitin from ubiquitinated proteins Spider biofilm induced
(orf19.4929) Ortholog(s) have mRNA binding, translation regulator activity and role in mitochondrial cytochrome c oxidase assembly, positive regulation of mitochondrial translational initiation
(orf19.1677) Protein of unknown function transcript detected on high-resolution tiling arrays
(orf19.1623) AP-1 bZIP transcription factor apoptotic, oxidative stress response/resistance, multidrug resistance nuclear in oxidative stress complements S. cerevisiae yap1 mutant oralpharyngeal candidasis-, human neutrop
(orf19.6826) Putative polysome-associated RNA binding protein macrophage-induced gene
(orf19.3888.2) Dubious open reading frame
(orf19.3888.2) Dubious open reading frame
Description
(orf19.1434) Ortholog(s) have DNA polymerase binding, protein kinase activator activity, signaling adaptor activity
(orf19.3694) Ortholog of <i>C. dubliniensis CD36</i> : Cd36_02210, <i>C. parapsilosis CDC317</i> : CPAR2_106350, <i>C. auris B8441</i> : B9J08_000959 and <i>Candida tenuis NRRL Y-1498</i> : CANTEDRAFT_116785
